# A mouse model of paralytic myelitis caused by enterovirus D68

**DOI:** 10.1371/journal.ppat.1006199

**Published:** 2017-02-23

**Authors:** Alison M. Hixon, Guixia Yu, J. Smith Leser, Shigeo Yagi, Penny Clarke, Charles Y. Chiu, Kenneth L. Tyler

**Affiliations:** 1 Medical Scientist Training Program, University of Colorado School of Medicine, Aurora, CO, United States of America; 2 Neuroscience Program, University of Colorado School of Medicine, Aurora, CO, United States of America; 3 Department of Laboratory Medicine and Medicine, Division of Infectious Diseases, University of California San Francisco, San Francisco, CA, United States of America; 4 UCSF-Abbott Viral Diagnostics and Discovery Center, University of California, San Francisco, San Francisco, CA, United States of America; 5 Department of Neurology, University of Colorado School of Medicine, Aurora, CO, United States of America; 6 California Department of Public Health, Richmond, CA, United States of America; 7 Denver VA Medical Center, Denver, CO, United States of America; 8 Departments of Immunology and Microbiology, and Medicine, University of Colorado School of Medicine, Aurora, CO, United States of America; University of Pittsburgh, UNITED STATES

## Abstract

In 2014, the United States experienced an epidemic of acute flaccid myelitis (AFM) cases in children coincident with a nationwide outbreak of enterovirus D68 (EV-D68) respiratory disease. Up to half of the 2014 AFM patients had EV-D68 RNA detected by RT-PCR in their respiratory secretions, although EV-D68 was only detected in cerebrospinal fluid (CSF) from one 2014 AFM patient. Given previously described molecular and epidemiologic associations between EV-D68 and AFM, we sought to develop an animal model by screening seven EV-D68 strains for the ability to induce neurological disease in neonatal mice. We found that four EV-D68 strains from the 2014 outbreak (out of five tested) produced a paralytic disease in mice resembling human AFM. The remaining 2014 strain, as well as 1962 prototype EV-D68 strains Fermon and Rhyne, did not produce, or rarely produced, paralysis in mice. In-depth examination of the paralysis caused by a representative 2014 strain, MO/14-18947, revealed infectious virus, virion particles, and viral genome in the spinal cords of paralyzed mice. Paralysis was elicited in mice following intramuscular, intracerebral, intraperitoneal, and intranasal infection, in descending frequency, and was associated with infection and loss of motor neurons in the anterior horns of spinal cord segments corresponding to paralyzed limbs. Virus isolated from spinal cords of infected mice transmitted disease when injected into naïve mice, fulfilling Koch’s postulates in this model. Finally, we found that EV-D68 immune sera, but not normal mouse sera, protected mice from development of paralysis and death when administered prior to viral challenge. These studies establish an experimental model to study EV-D68-induced myelitis and to better understand disease pathogenesis and develop potential therapies.

## Author summary

Reports of polio-like paralysis, referred to as acute flaccid myelitis (AFM), have recently emerged in association with infections caused by enterovirus D68 (EV-D68). In the second half of 2014, 120 cases of AFM, mostly in young children, were reported during a nationwide outbreak of EV-D68 respiratory disease. The number of AFM cases has risen again in 2016. Although epidemiological evidence between EV-D68 infection and AFM is accumulating, a causal link has not been definitely established. Here we demonstrate that strains of EV-D68 recovered during the 2014 epidemic can cause a paralytic illness in mice that resembles human AFM. Evidence that EV-D68 causes paralysis in this mouse model include: (1) loss of spinal cord motor neurons innervating paralyzed limbs, (2) detection of virus in the spinal cord and, specifically, motor neurons, (3) transmission of neurological disease when injecting virus isolated from spinal cords of paralyzed mice into naïve mice, thus fulfilling Koch’s postulates, and (4) the ability to prevent AFM by pre-administering serum containing EV-D68 antibodies from previously infected mice. This experimental mouse model can be used to better understand the pathogenesis of EV-D68-induced CNS disease and to facilitate the development of potential therapies.

## Introduction

Enterovirus D68 (EV-D68) was first identified in 1962 after it was isolated from four children in California with acute respiratory illnesses [1]. Enteroviruses are typically spread through fecal-oral transmission, and are associated with diarrheal illnesses, undifferentiated fever with rash, hand-foot-and-mouse disease, meningitis, and encephalitis [2]. EV-D68, however, possesses several properties more similar to rhinoviruses than conventional enteroviruses, including optimal replication in the cooler temperatures of the upper respiratory tract (33°C), acid sensitivity, and spread primarily via respiratory, rather than fecal-oral, transmission [1, 3, 4].

Passive surveillance data from the National Enterovirus Surveillance System (NESS) indicated that EV-D68 has been a rare cause of respiratory illness, with only 26 documented cases of EV-D68 in the United States (US) from 1970–2005 [5]. However, within the last decade, EV-D68 outbreaks have become more common worldwide [6, 7], and in the second half of 2014, the US experienced an unprecedented EV-D68 respiratory disease outbreak with over 1150 cases reported nationwide [8–10]. This number is almost certainly an underestimate, as only the most severe cases underwent pathogen identification, and rapid tools for laboratory confirmation of EV-D68 in the clinical setting were not widely available until mid-2015 [11].

Coincident with the US EV-D68 respiratory outbreak, physicians began reporting an increased number of cases of acute flaccid paralysis with a striking resemblance to poliomyelitis [12–16]. The paralysis occurred primarily in young children (median age ~7) [17–20]. Many children experienced prodromal symptoms of fever and upper respiratory illness before the onset of limb weakness [14, 16–21]. Magnetic resonance imaging (MRI) showed signal abnormalities in the anterior horns of the spinal cord, the location of motor neurons innervating upper and lower limbs [14, 16, 19–21]. The CDC established guidelines for reporting acute flaccid myelitis (AFM), defined as acute limb weakness with characteristic spinal cord imaging abnormalities on MRI occurring in children on or after August 2014. Under these guidelines, 120 confirmed cases of AFM from 34 states were documented in 2014 [17]. Sporadic cases meeting the CDC definition, from 0 to 7 per month, continued to be reported in 2015, and in 2016 the CDC reported 132 confirmed cases of AFM from 37 states. [17]. Case-control study data from the 2014 cluster of AFM cases in Colorado showed that the odds of a child with AFM having had a concomitant EV-D68 respiratory infection were over 10 times greater than for controls with acute respiratory disease [22]. EV-D68 RNA was detected in respiratory secretions as the predominant pathogen in about half of the affected children tested in 2014, although EV-D68 RNA was amplified from cerebrospinal fluid from only one 2014 AFM patient to date [18, 19]. There was no statistical correlation of AFM with infection by any other pathogen in respiratory samples [22], and in-depth metagenomic sequencing of CSF failed to reveal an alternative infectious etiology [18].

Given the lack of consistent CSF isolation, a potential causal role of EV-D68 in AFM has not been formally established. Several additional pieces of evidence, however, suggest that EV-D68 has the capacity to produce central nervous system (CNS) disease. Other viruses in the *Enterovirus* genus, such as polioviruses, enterovirus 70, and enterovirus 71, are established causes of acute flaccid paralysis. EV-D68 has also been described as a cause of neurological disease in two previous case reports [5, 23]. The first involved a young adult who developed acute flaccid paralysis with EV-D68 detected in CSF in 2005 [5]. The second involved a 5-year-old boy who developed fatal EV-D68 meningomyeloencephalitis with neuron loss in motor nuclei of the brain and cervical spinal cord in 2008 [23].

Changes in the viral genome can also potentially contribute to virulence and spread. EV-D68 strains have undergone significant evolutionary shifts since their original isolation [7, 18]. The Fermon and Rhyne strains, obtained from respiratory swabs of children in California in 1962, are considered the prototype EV-D68 strains [1]. During the mid-1990’s, the prototype lineage separated into two clades, designated A and C [7]. Clade B further separated from clade C in the mid-2000s [7]. A clade named B1 then separated from clade B around 2010 [18]. Most of the EV-D68 respiratory cases that occurred in the 2014 epidemic were caused by a single lineage of clade B1 viruses related to strains previously seen circulating in the US, Asia, and southern Europe from 2011–2013 [9]. A minority of cases were caused by clade A and C members [9]. Of the AFM patients analyzed in the United States, up to half had respiratory sputum samples positive by reverse transcription polymerase chain reaction (RT-PCR) for EV-D68, with increasing likelihood of EV-D68 detection if the sample was collected closer to the onset of prodromal febrile/respiratory symptoms [18, 19]. Metagenomic sequencing of available EV-D68 positive respiratory samples in one study from California and Colorado mapped all AFM-associated EV-D68 strains to clade B1 [18]. Because non-clade B1 strains were less prevalent, however, it is possible this study was unable to detect an association between AFM and other clades.

In order to develop an animal model of EV-D68-associated neuropathogenesis, we screened both 2014 outbreak and prototype 1962 EV-D68 strains for the ability produce paralysis in mice. The 2014 strains tested were isolated from clinical respiratory samples from the 2014 outbreak and represented clades A, B, and B1.

## Results

### Contemporary EV-D68 strains cause limb paralysis in neonatal mice

**Fig 1:**
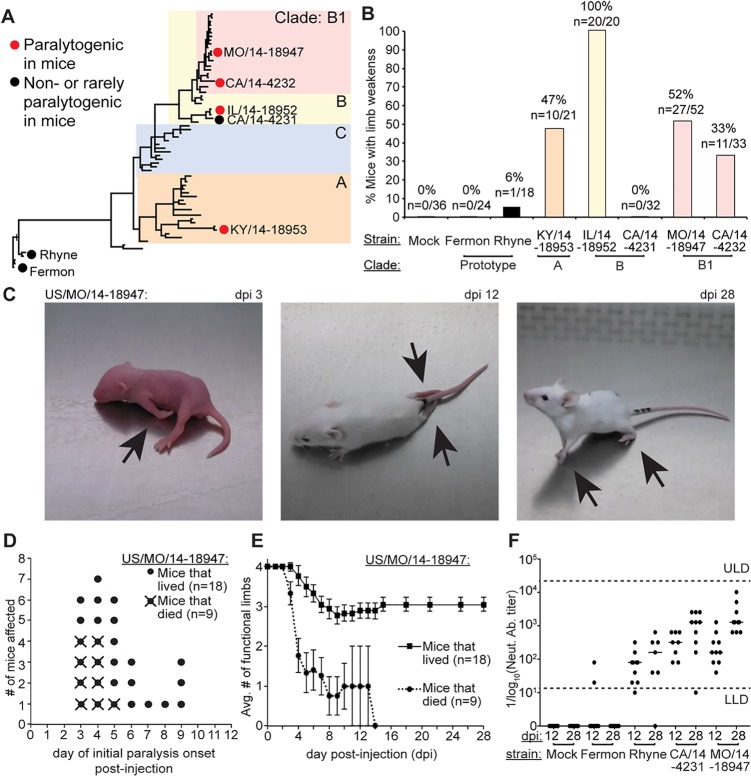
EV-D68 causes limb paralysis in neonatal mice. (**A**) Phylogenetic tree of EV-D68 based on VP1 sequence showing each strain tested in this study. (**B**) Bar graph showing the percent of mice with paralysis following intracerebral injection with each EV-D68 strain or control media. (**C**) Examples of limb paralysis (arrows) in 3 different mice following intracerebral injection of MO/14-18947. Left–day post-infection 3 (dpi 3), center–dpi 12, right–dpi 28. (**D**) Day of paralysis onset in mice after intracerebral injection with MO/14-18947. Mice that died (n = 9 circles with “X”) generally had earlier onset of paralysis than mice that lived (n = 18 circle). (**E**) Average number of functional (non-paralyzed) limbs in mice following intracerebral injection with MO/14-18947 in mice that lived (solid line, squares) and mice that died (dotted line, circles). Error bars represent the standard error of the mean (SEM). (**F**) Serum anti-EV-D68 neutralizing antibody titer in mice (reciprocal serum dilution) following intracerebral injection with MO/14-18947 at dpi 12 and dpi 28 (n = 10), Fermon (n = 8–10), Rhyne (n = 7–10), CA/14-4231 (n = 8–10) or mock (n = 8). The line in each group on the graph represents the median titer. Lower limit of assay detection (LLD) of 1:10 and upper limit of assay detection (ULD) of 1:10,240 are indicated by the dotted lines.

Five EV-D68 strains from the 2014 outbreak (clade A strain KY/14-18953; clade B strains IL/14-18952 and CA/14-4231; B1 strains MO/14-18947 and CA/14-4232) and two prototype strains (Fermon and Rhyne) were screened for the ability to cause neurological disease in two day-old outbred Swiss Webster mice (Fig 1A and 1B). Intracerebral injection of viral strains was chosen to investigate neurovirulence and neurotropism by bypassing potential barriers to viral central nervous system (CNS) entry. Strains were injected at the highest available viral titer to maximize the possibility of eliciting disease (see Methods). Mice were monitored daily for up to 4 weeks or until death occurred.

Following injection, four out of the five contemporary strains tested (KY/14-18953, IL/14-18952, MO/14-18947, and CA/14-4232) induced paralytic disease in 33–100% of mice (Fig 1B). Paralysis occurred in these mice between dpi 3 –dpi 9 for all strains. Mortality (5–70%) varied by strain, and most of the mice that died had paralysis (31 out of 38 deaths, 80%). Example images of mice injected with one of these strains, MO/14-18947, can be seen in Fig 1C and S1–S3 Movies. One contemporary strain, CA/14-4231, failed to induce paralysis or death despite its close phylogenetic relationship to IL/14-18952 (Fig 1A and 1B).

The degree of limb involvement ranged from monoparesis to quadriparesis. Paralysis in very young mice (postnatal days 5–7) could be identified as a reduction of movement in one or more limbs, leading to a reduced ability to crawl and turn when evaluated on a flat surface (Fig 1C, left panel; S1 Movie). In older, more mobile mice (~8 days old or more), paralysis could be assessed along a continuum from mild loss of motor function, as exemplified by toe or knuckle walking, to the complete inability to use the limb for ambulation (Fig 1C, center and right panels; S2 and S3 Movies). Affected limbs appeared to hang in unnatural positions and developed atrophy over time. Sensation in affected limbs remained grossly intact as determined by response of vocalization and attempt to move away from the noxious stimuli (toe pinch test) [24].

**Fig 2:**
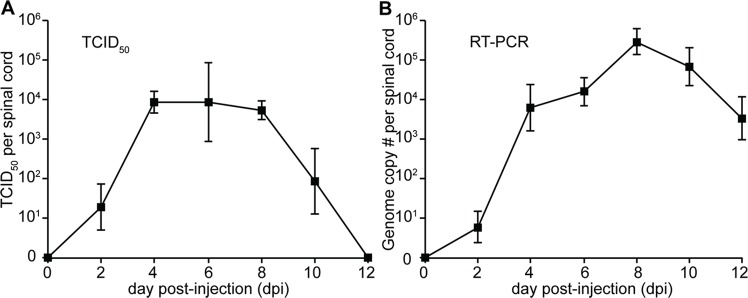
Infectious virus and EV-D68 RNA can be detected in the spinal cords of infected mice. (**A**) Graph shows the mean TCID_50_ titer in spinal cords from mice infected intracerabrally with MO/14-18947. Between 5 and 11 mice from up to 2 litters were collected for each time point. At dpi 0 virus was undetectable in the spinal cords of infected mice, but mean viral titer rose steadily at dpi 2, although the mice in this group had not yet developed signs of paralysis. Paralyzed mice examined on dpi 4 showed further increase in mean spinal cord viral titer. Mean spinal cord titer plateaued from dpi 4 through dpi, 8 and then declined, becoming undetectable by dpi 12 (**B**) Mean genome copy number in whole spinal cords from the same mice in (A) quantified by two-step RT-PCR targeting EV-D68 VP1 paralleled the titers seen in (A), although viral genome remained detectable on dpi 12 despite absence of infectious virus. This indicates that viral genome can remain detectable in tissue longer than viable infectious particles. Note that estimated genome copies were used as relative comparisons between each time point and are likely an underestimate of absolute viral RNA due to inefficiency of RNA extraction from animal tissue and amplification by two-step RT-PCR. Error bars represent SEM.

Compared to the contemporary strains, mice injected with the prototype strains, Rhyne and Fermon, had fewer signs of morbidity or mortality. One mouse in the Rhyne group (n = 1 out of 18, 6%) developed a transient right hindlimb weakness that was obvious only during ambulation (Fig 1B). Signs of weakness began on dpi 5 but disappeared by dpi 9. The mouse was able to sit with this limb in proper position while at rest, and the limb did not show signs of atrophy. Rhyne has been reported to cause a myositis [1], and this may have accounted for the weakness noted. Mice injected with either Fermon (n = 24) or rhabdomyosarcoma (RD) cell culture media (n = 36) showed no evidence of paralysis (Fig 2B). Two mice, one in the Fermon group and one in the Rhyne group, died early (dpi 2–4), although they did not show signs of neurological disease or paralysis prior to death. The cause of death in these two mice was unclear.

One strain from clade B1, MO/14-18947, was chosen for further in-depth analysis of the paralysis phenotype. MO/14-18947 was chosen because it belongs to clade B1, the predominant circulating EV-D68 clade in 2014. In mice infected with MO/14-18947, the onset of paralysis occurred most frequently between dpi 3–5, although onset occasionally occurred as late as dpi 9 (Fig 1D). Mice that developed earlier paralysis were more likely to die than mice that developed paralysis at a later age, with an overall death rate in paralyzed mice of 33%. Mice dying of infection had more severe paralysis, as defined by the average number of limbs affected, compared to mice surviving infection (Fig 1E).

Following intracerebral injection of the virus, forelimbs were most commonly affected at paralysis onset (52%), although some mice presented with hindlimb paralysis (30%) or both forelimbs and hindlimbs affected (18%). Up to 48% of mice experienced disease progression to other limbs after onset of initial paralysis (Fig 1E). The majority (72%) of mice surviving to dpi 28 showed no motor recovery as quantified by the number of limbs affected; the others (28%) recovered between dpi 9 to 15. Recovery occurred most often in mice with milder disease (e.g. only one limb affected) (Fig 1E).

Mice injected with MO/14-18947 generated neutralizing antibody titers against EV-D68, regardless of the presence or absence of paralysis. Sera of mice tested at dpi 12 (n = 12) had antibody titers ranging from 1:40 to 1:1,280. Neutralizing antibody titer increased in MO/14-18947-infected mice tested at dpi 28 (n = 11), with a titer range of 1:640 to 1:>10,240 (Fig 1F). Neutralizing titers in MO/14-18947 mice were compared to those in non-paralytogenic strains in order to determine whether these strains were able to evoke a serological response in the host (Fig 1F). Fermon frequently failed to generate a detectable antibody response in infected mice. Only two mice out of 10 mice tested at dpi 12 developed neutralizing antibodies with titers ranging from 1:20–1:80, whereas none of the Fermon-infected mice tested at dpi 28 (n = 18) had detectable neutralizing titers. In contrast, mice infected with Rhyne (n = 7–10) and CA/14-4231 (n = 8–10) both generated a sustained neutralizing antibody response against their respective strains, similar to those seen in mice infected with MO/14-18947 (Fig 1F). Mice injected with media control did not have detectable anti-EV-D68 antibody titers at either dpi 12 (n = 8) or dpi 28 (n = 8). To confirm EV-D68 infection of these strains in mice, viral titers were examined in the skeletal muscle after intramuscular inoculation. All strains examined, except Fermon, replicated to equivalent viral titers in the muscle regardless of the ability to induce paralysis. Fermon did not replicate and did not cause paralysis. The ability to infect skeletal muscle corresponded to the ability to produce neutralizing antibodies (S1 Table).

### EV-D68 spinal cord titer corresponds to paralysis onset and disease progression

Viral growth in spinal cords from groups of paralyzed mice injected intracerebrally with EV-D68 MO/14-18947, as well as from litters of mice tested before the typical onset of paralysis (dpi 0 and dpi 2), was quantified by TCID_50_ assay (Fig 2A). Infectious virus was not detected in the spinal cords of any mice on dpi 0, but mean viral titer increased progressively in the spinal cords of mice tested on dpi 2 and dpi 4. The mean spinal cord titer then remained >1000 TCID_50_ in mice tested through dpi 8, after which it dropped steadily and became undetectable by dpi 12. Two-step quantitative RT-PCR (qRT-PCR) for EV-D68 utilizing primers targeting the VP1 capsid gene was used to detect EV-D68 RNA extracted from whole spinal tissue from mice from each time point post-infection (20). Results of the qRT-PCR analysis paralleled the results of the TCID_50_ assay, although titer by qRT-PCR remained detectable at dpi 12, indicating a longer time of detectability of viral genome as compared to infectious particles in tissue (Fig 2B).

Infectious virus was detected in the brains of dpi 0 mice by TCID_50_ assay approximately an hour after injection (average 10^2+/-1.05^ TCID_50_/mL). However, by dpi 2 the virus could only be detected in brains from 2 out of 11 mice by TCID_50_ assay, and from dpi 4 to 12 no virus was detected in brain of any paralyzed animals. Infectious virus remained below the limits of quantification by TCID_50_ assay in sera from all paralyzed mice.

### EV-D68 infects motor neurons and results in motor neuron death

**Fig 3:**
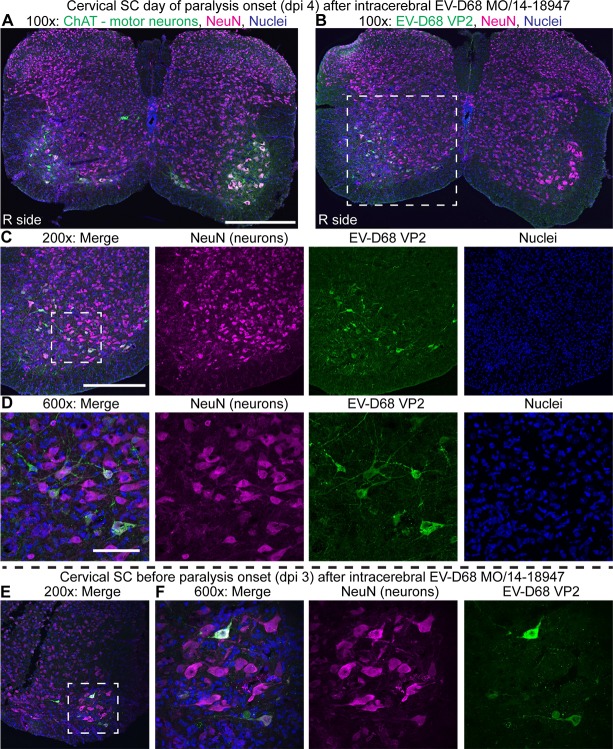
EV-D68 infects anterior horn motor neurons and results in motor neuron death. (**A**) A cervical spinal cord section at 100X original magnification from a mouse injected intracerebrally with EV-D68 MO/14-18947 that developed right forelimb paralysis on day 4 post-injection. Loss of motor neurons (green, labeled with choline acetyltransferase / ChAT) is observed in the right (“R side”) anterior horn, corresponding to the affected side. In contrast, motor neurons of the left anterior horn corresponding to the unaffected side are relatively intact. (**B**) A consecutive cervical spinal cord section from the same dpi 4 mouse at 100X original magnification reveals the presence of EV-D68 VP2 capsid protein within the few remaining right anterior horn neurons. The box represents the area imaged at 200X in (C). (**C**) 200X and (**D**) 600X images of the right anterior horn stained for EV-D68 VP2 (green). The box represents the area imaged at 600X in (D). (**E**) 200X and (**F**) 600X images from a left anterior horn in an intracerebral-injected mouse at day 3 post-injection before the onset of paralysis showing EV-D68 antigen in an intact cluster of motor neurons. The box represents the area imaged at 600X in (F). For all images, neurons (magenta) are labeled with NeuN, a general neuron marker, and nuclei (blue) are labeled with Hoechst 33342. Scale bars for 100X original magnification are 400 μm, 200X are 200 μm, 600X are 50 μm.

**Fig 4:**
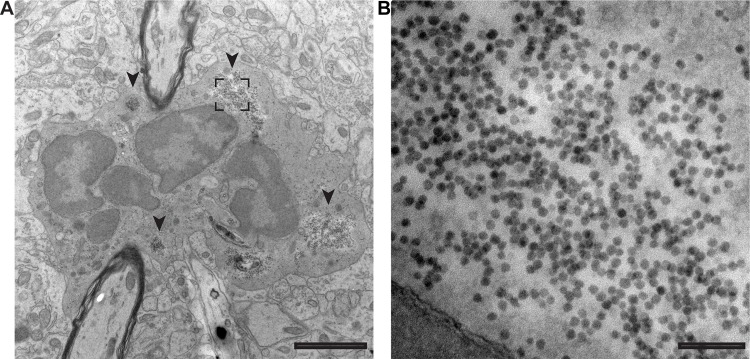
Large numbers of particles morphologically consistent with enterovirus are seen in dying anterior horn cells of the spinal cord following intracerebral injection of EV-D68. TEM images were taken from the cervical spinal cord anterior horn of a day 4 post-injection MO/14-18947 mouse with new onset forelimb paralysis. (**A**) A dying cell, consistent in position and morphology with a motor neuron, showing nuclear fragmentation, cytoplasmic blebbing, and regions dense with particles morphologically consistent with enteroviruses (arrowheads). The box represents the area imaged in (B). (**B**) A higher magnification image shows a cluster of particles morphologically consistent with enteroviruses. Scale bars are 2 μm and 500 nm for low (11,000X) and high magnification (98,000X), respectively.

Pathological examination of mice infected intracerebrally with MO/14-18947 revealed marked injury and loss of motor neuron populations in the anterior horn of the spinal cord corresponding to the ipsilateral affected limb (Fig 3). Fig 3 illustrates a typical case with marked injury and loss of the motor neuron population in the anterior horn ipsilateral to the affected right limb as indicated by loss of choline acetyltransferase (ChAT) staining, a specific marker of spinal cord anterior horn motor neurons, and NeuN staining, a general marker of neurons (Fig 3A). In contrast, the motor neuron population corresponding to the unaffected left limb appeared intact (Fig 3A). Examination of a consecutive spinal cord section stained for EV-D68 VP2 capsid protein revealed viral antigen within the few remaining motor neurons on the affected side (Fig 3B–3D). No staining was seen on the unaffected side (Fig 3B) or in media injected control mice (S1 Fig). To demonstrate the presence of virus at an earlier time point following infection (during which the motor neuron population was relatively intact), spinal cords from dpi 3 mice from a litter injected intracerebrally with MO/14-18947 that had not yet begun to show signs of paralysis were examined. EV-D68 VP2 antigen was consistently detected within motor neurons in these mice by immunostaining (Fig 3E). Unfortunately, direct co-localization of EV-D68 antigen and ChAT was not obtainable due to inability to find compatible primary antibodies from different host species for co-labeling, so consecutive sections (10 um apart) were used for these staining experiments. Transmission electron microscopy (TEM) images of the cervical spinal cord anterior horn from a dpi 4 MO/14-18947 injected mouse with forelimb paralysis confirmed the presence of dying cells, consistent in location and morphology with motor neurons, filled with cytoplasmic clusters of ~30 nm particles morphologically consistent with enteroviruses (Fig 4A and 4B) [25].

### Alternative routes of EV-D68 infection produce paralytic disease

**Fig 5:**
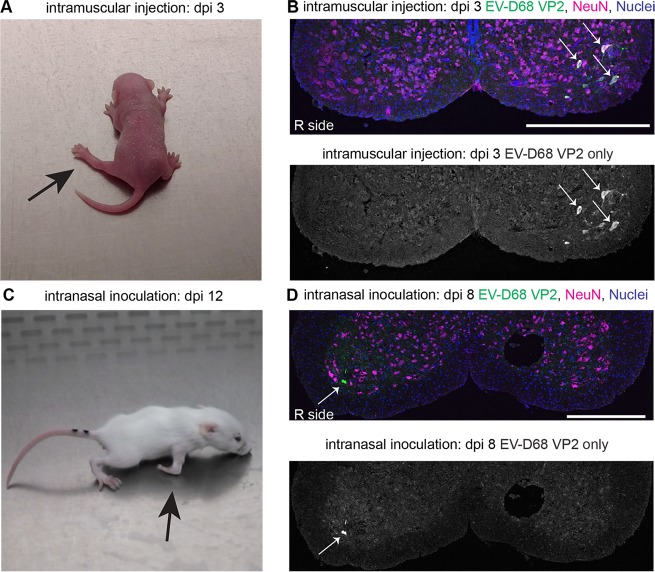
EV-D68 causes paralysis after intranasal and intramuscular infection. (**A**) An image of a day 3 post-infection mouse with new onset left hindlimb paralysis following intramuscular injection of EV-D68 strain MO/14-18947. (**B**) Lumbar spinal cord sections (100X original magnification) from an intramuscular-injected mouse at day 3 post-infection with left hindlimb paralysis showing loss of motor neurons and viral antigen (arrows) in the left anterior horn. (**C**) An image of a mouse on day 12 post-infection that developed right forelimb paralysis following intranasal infection with EV-D68 strain MO/14-18947. (**D**) Cervical spinal cord sections (100X original magnification) from a day 8 post-infection intranasal-infected mouse with right forelimb paralysis showing viral antigen in the anterior horn (white arrow). The scale bars are 400 μm. Abbreviation: dpi, days post-infection.

In order to determine whether routes of EV-D68 infection other than intracerebral injection could produce paralytic disease, mice were infected intramuscularly, intranasally, or intraperitoneally with the MO/14-18947 strain. Intramuscular injection of EV-D68 into the left hindlimb produced paralysis in 100% (n = 18 out of 18, 100%) of injected animals. Paralysis onset occurred from dpi 2 to 3 in the injected hindlimb before often progressing to the contralateral hindlimb and then forelimb(s) (Fig 5A, S4 Movie). EV-D68 RNA in the spinal cord was detected by RT-PCR in additional mice (n = 5 out of 5, 100%) examined on dpi 3 following left hindlimb injection, with an average estimated copy number per spinal cord of 10^5.3±0.7^. Examination of the spinal cords from dpi 3 mice injected into the left hindlimb revealed infection of motor neurons and viral antigen consistent with signs of paralysis (Fig 5B). An additional group of mice (n = 8 out of 8) injected with MO/14-18947 in the right forelimb also developed paralysis starting in the injected limb between dpi 2–4, consistent with the onset of paralysis seen in the hindlimb injections. In contrast, intramuscular injection with RD control media failed to produce paralysis (n = 0 out of 14, 0%).

Although rare, paralysis following intranasal infection was observed in 2 of 73 mice (2.7%) of mice showing signs of paralytic disease. Onset of paralysis occurred in these mice between dpi 8 and 10. One mouse developed paralysis in the right forelimb only (Fig 5C; S5 Movie); the other had mild left forelimb paralysis and appeared generally weak in all limbs. The mouse with paralysis onset at dpi 10 was sacrificed on dpi 12 (Fig 5C), and examined for the presence of EV-D68 RNA in its spinal cord tissue by RT-PCR. It was found to be positive for EV-D68 in its spinal cord tissue with an estimated EV-D68 genome copy number of 10^4.0^, comparable to viral titers found in mice after intracerebral injection. The other mouse that developed signs of paralysis after intranasal infection was sacrificed for histological examination on dpi 8 and showed viral antigen in the motor neurons of the cervical spinal cord (Fig 5D).

Intraperitoneal injection of MO/14-18947 produced disease in only 1 out of 22 (n = 4.5%) mice. Paralysis in this mouse occurred in the right rear leg on dpi 5.

### Koch’s postulates are fulfilled for the mouse model of EV-D68 paralytic disease

**Fig 6:**
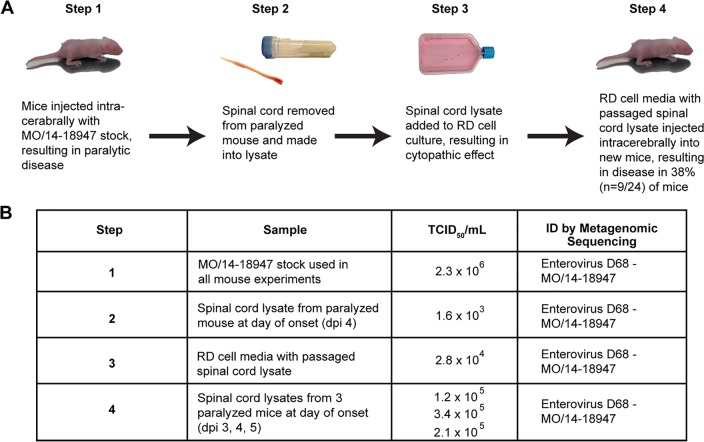
Fulfillment of Koch’s postulates establishes a causal role for EV-D68 in paralytic disease. (**A**) Steps taken to fulfill Koch’s postulates in the EV-D68 mouse model of AFM. Mice were infected with MO/14-18947 by intracerebral injection and monitored daily until the onset of paralysis (Step 1). The whole spinal cord of a paralyzed mouse was removed and mechanically lysed (Step 2). The spinal cord lysate was then inoculated into a flask of RD cells (Step 3). After the appearance of cytopathic effect, the RD cell media with passaged spinal cord lysate was collected and syringe filtered. The filtered media with passaged spinal cord lysate was then injected intracerebrally into naïve mice (Step 4). 38% (n = 9 out of 24) of these mice developed signs of paralysis. Spinal cords from several paralyzed mice (n = 3 out of 3) in Step 4 were removed and examined by TCID_50_ and metagenomic sequencing for the presence of EV-D68. (**B**) TCID_50_ analysis revealed infectious virus confirmed to be MO/14-18947 by metagenomic deep sequencing identification for each step in the fulfillment of Koch’s postulates.

To determine whether EV-D68 was the direct cause of the paralytic disease in mice by fulfilling Koch’s postulates [26], spinal cord lysate from a dpi 4 mouse with signs of paralysis following intracerebral injection of MO/14-18947 was cultured in rhabdomyosarcoma (RD) cells. Significant cytopathic effect (CPE) was noted within 3 days in the inoculated RD cell culture. The spinal cord-passaged cell culture lysate was passed through a 0.22 μM filter syringe and injected intracerebrally into the brains of naïve mice (Fig 6A). 38% (n = 9 out of 24) injected with the cell culture lysate developed paralytic disease between dpi 3 and 8. This rate of paralysis is consistent with that seen in previous experiments (e.g., Fig 1B), especially when considering that the inoculum was nearly 100-fold lower than that used in other experiments (TCID_50_ 10^4^/mL compared with TCID_50_ 10^6^/mL). Analysis of 3 mice that developed early severe paralysis revealed high spinal cord viral titers by TCID_50_ (Fig 6B). Metagenomic deep sequencing followed by SURPI (sequence-based ultra-rapid pathogen identification) analysis confirmed the presence of EV-D68 strain MO/14-18947 RNA in the original spinal cord lysate, the RD cell culture lysate, and the spinal cord tissue of the mice injected with the spinal cord-passaged cell culture lysate (Fig 6B; Tables A-D in S1 Text) [27]. No paralysis or other signs of disease were seen in mice (n = 12) injected with RD cell culture lysates that had been inoculated with normal mouse spinal cord.

### Passive anti-EV-D68 antibody transfer prevents paralytic disease and death

To evaluate the role of immune sera in protection against disease, 1 day-old mice were injected intraperitoneally with either pooled immune sera against MO/14-18947 (neutralizing antibody titers: 1:320–640) or pooled control normal mouse sera (neutralizing antibody titer undetectable) and then challenged 24 hrs later with intracerebral injection of MO/14-18947 (2.3 x 10^6^ TCID_50_/mL). 57% of mice (n = 12 out of 21) receiving normal mouse sera developed paralysis compared to only 4.5% of mice (n = 1 out of 22) treated with EV-D68 immune sera (*p* < 0.0002 by Fisher’s Exact Test). There were no deaths in the immune sera treated group and 18% mortality in mice receiving normal mouse sera (*p* = 0.05 by Fisher’s Exact Test).

## Discussion

We have described a mouse model of spinal cord infection and paralysis caused by clinical isolates of EV-D68. Of five 2014 EV-D68 strains tested, four strains induced a paralytic disease in mice following intracerebral injection. An in-depth characterization of one of these strains, clade B1 MO/14-18947, revealed that this paralysis replicated key features of human AFM including a lower motor neuron pattern of paralysis with a predilection for the upper limbs, limited motor recovery over time, and no sensory or cerebral involvement [13, 16, 19, 20]. MRI studies and electromyography of AFM patients suggest loss of motor neurons without damage to sensory pathways (12, 17, 24). Consistent with these findings, paralyzed mice exhibited no gross loss of sensory function, and viral antigen was detected almost exclusively in cells consistent with motor neurons as determined by ChAT staining, morphology, and anatomical location within the spinal cord. EV-D68 appears to have a specific tropism for spinal cord motor neurons as demonstrated by the absence of significant growth in brain and the restricted pattern of antigen distribution and neuronal injury in the spinal cord. The kinetics of viral growth closely paralleled the observed development of paralysis, suggesting that direct viral injury, rather than a post-infectious immune-mediated process is the most likely mechanism of neuronal cell loss and subsequent paralysis. Fulfillment of Koch’s postulates supports a causal role of EV-D68 infection in the development of paralytic disease in this model.

We also established that EV-D68 neuroinvasion could occur by several alternative routes in addition to producing disease after intracerebral injection. Although intranasal infection rarely produced paralytic disease, mice that developed signs of paralysis exhibited spinal cord infection similar to that seen following other routes of infection. The rarity of paralytic disease following intranasal infection (~3%) of EV-D68 is consistent with the low incidence of AFM after EV-D68 respiratory infection in humans (likely <1%). Surprisingly, intramuscular infection produced paralysis even more consistently than intracerebral infection. As EV-D68 appears to have specific tropism for motor neurons, we hypothesize that intramuscular infection may be a more efficient and direct pathway to motor neuron infection than intracerebral injection.

The exact pathways and mechanism of spread for EV-D68 after virus inoculation at specific sites (intramuscular, intracerebral, or intranasal) remain to be fully elucidated. In the current study, mice injected intramuscularly in either the forelimb or the hindlimb initially developed paralysis in the inoculated limb. Similarly, the intranasally inoculated mice and the majority of the intracerebrally inoculated mice initially developed forelimb paralysis. This pattern of paralysis onset after different routes of inoculation is most consistent with viruses that spread along neural pathways. Neural spread typically involves initial infection of and injury to the segments of the spinal cord containing neurons innervating the site of inoculation [28]. In contrast, viremic spread characteristically results in uniform involvement of motor neurons innervating forelimbs and hindlimbs, regardless of the site of inoculation [28]. The rarity of paralysis following intraperitoneal inoculation (a proxy for intravenous inoculation) and the lack of infectious virus in sera of paralyzed mice in this model argue against a critical role for viremic spread in neuropathogenesis. Notably, EV-D68 viremia has only been rarely identified in human AFM patients (1 in 25, or 4%, as reported in one study) [18]. Ultimately, several mechanisms may facilitate spread (neural or viremic) depending on the route of infection, as have been described for polioviruses [29].

To date, only EV-D68 strains from clade B1 have been isolated from AFM patients [18]. A previous study identified six polymorphisms confined to AFM-associated clade B1 strains that could represent genetic changes associated with neurovirulence [18]. However, in the current study, strains from multiple clades (A, B, and B1) produced paralysis in neonatal mice (a 2014 clade C strain was not available for testing) (S2 Fig). These data indicate that the clade B1-specific polymorphisms and sequence homology alone do not fully explain paralysis in this mouse model. In addition, the observed molecular epidemiologic association of clade B1 with AFM may be due to the high overall prevalence of this clade circulating in the population in 2014 [18]. Unexpectedly, one contemporary clade B strain, CA/14-4231, failed to induce paralysis, despite its close phylogenetic relationship to a neurovirulent clade B strain, IL/14-18952 (S2 Fig). Failure to induce paralysis was not a result of the inability to infect mice (S1 Table), suggesting that CA/14-4231 may lack genetic sequences critical for neurovirulence. Further comparative analyses using infectious clones will likely to be needed to establish the determinants of neurovirulence and host range of EV-D68 in mice. It would also be informative to test EV-D68 strains from Europe, North America, and Africa associated with smaller outbreaks in 2009/2010 (clades A, B, and C), but these were not available for the current study [7, 30]. Furthermore, population factors, such as spread in a large immunologically naïve population and host-specific genetic susceptibility, also cannot be fully ruled out as contributors to AFM. The previous finding of a sibling pair, both infected by identical strains of a clade B1 EV-D68 strain, yet only one developing AFM [18, 19], points to the potential importance of such host-related factors in viral neuropathogenesis.

Finally, a mouse model is an important first step in the *in vivo* screening of potential drug and vaccine therapies against EV-D68, for which there are currently no established treatments. In the current study, treatment of naïve neonatal mice with EV-D68 immune sera containing anti-EV-D68 antibodies, but not normal mouse sera, prevented paralysis and death after viral challenge. Additional mouse studies testing specific antiviral agents or delaying passive antibody administration until later in the disease course are currently in progress. The results presented here suggest that immunomodulatory strategies such as vaccination or the use of EV-D68 hyperimmune sera may be potentially effective strategies for treatment or prevention of EV-D68 associated neurological disease. Our findings are of particular relevance given the recent surge in AFM cases in 2016 [17].

## Materials and methods

### Ethics statement

All studies were done in accordance with the University of Colorado IACUC and Animal Use Committee (B-34716(03)1E). Mice were cared for in adherence to the NIH Guide to the Care and Use of Laboratory Mice. Mouse pups exhibiting paralysis were euthanized if unable to nurse. Mice were anaesthetized with inhaled isoflurane before tissue collection or perfusion.

### Viral stock preparation

All EV-D68 viral strains were obtained from the American Type Culture Collection (ATCC) or from the California Department of Public Health (courtesy of Shigeo Yagi). Viral stocks were grown in RD cells (ATCC) at 33°C and 5% CO_2_ until most cells were dead or dying. Cells debris was removed from RD grown stocks by ultracentrifugation. Titers of viral stocks were determined by TCID_50_ assay as calculated by the Kärber method. The TCID_50_/mL titers for each strain used in this paper are as follows: Fermon– 8x10^6^, Rhyne– 1x10^7^, MO/14-18947 (clade B1)– 5x10^6^, CA/14-4232 (clade B1)– 1x10^5^, IL/14-18952 (clade B)– 5x10^7^, CA/14-4231 (clade B)– 3x10^7^, KY/14-18953 (clade A)– 2x10^6^.

The pure culture stock used in the Koch’s postulate experiment was grown from whole spinal cord lysate on RD cells. The stock was passed through a 0.22-micron sterile syringe filter before injection.

### Mouse experiments

All experiments were performed on NIH Swiss Webster mouse pups of both sexes from Envigo (Indianapolis, IN). Mouse litters were randomly assigned to experimental groups. Unless otherwise specified, virus infections were performed on two day-old mice. For intracerebral infection, mice were injected with ~20 μL via insulin syringe (29 G needle) with undiluted virus or RD control media stock into the right hemisphere just anterior to lamboid suture. For intramuscular infection, mice were injected with ~20 μL via insulin syringe (29 G needle) with undiluted virus into the medial aspect of the left hindlimb. For intraperitoneal infection, mice were injected with ~20 μL via insulin syringe (29 G needle) with undiluted virus into the peritoneal cavity. For intranasal infection, a total of 40 μL of undiluted virus was micro-pipetted onto the noses of a post-natal day 2 pups in two boluses of 20 μL with a 30-minute interval between exposures. Mice were examined daily for signs of paralysis. For passive transfer experiments, 100 μL of pooled sera was given by intraperitoneal injection via insulin syringe on post-natal day 1 to mice randomized between two litters. Immune sera was pooled from dpi 28 mice (n = 12) previously injected intracerebrally with US/MO/14-18947. Control sera were pooled from dpi 28 mice (n = 12) previously injected intracerebrally with RD control media. The passive antibody transfer experiment data is displayed as a combination of two replicate experiments. Mice were randomized between control and experimental conditions for each replicate. In all studies, no mice were excluded from analyses.

### Tissue collection and analysis

Serum was collected for TCID_50_ analysis and neutralizing antibody titer analysis. After collection, whole blood was placed immediately on ice, spun at 14,000 rpm for 10 minutes at 4°C, and then the serum was removed and placed in a fresh tube. Serum was stored at -20°C if used for antibody neutralization or at -80°C if used for TCID_50_ analysis. Brains were removed intact and placed in a BeadBug tissue homogenizer tubes (Benchmark Scientific, Edison, NJ) with 3.0 mm beads and brought to a standard volume of 1 mL with PBS. Spinal cords were removed intact as previously described and placed in a BeadBug tissue homogenizer tubes with 3.0 mm beads and brought to a standard volume of 0.3 mL with PBS [32]. Muscle tissue (anterior and posterior lower leg muscles) was removed and weighed, and it was then brought to a standard volume of 0.3 mL of PBS. Tissues were then mechanically lysed in the BeadBug microtube homogenizer (Benchmark Scientific, Edison, NJ). 60 μL of each tissue was removed for TCID_50_ analysis. Titer of virus in each tissue was determined by TCID_50_ assay and final viral titer was calculated using the Kärber method. Lack of growth below the detectable limit was graphed as a titer of zero.

For RT-PCR, total RNA from the remaining spinal cord sample was extracted using a Qiagen RNeasy Plus Micro Kit with RNA carrier (Hilden, GER) to facilitate RNA pull-down from this small tissue. Total RNA from each spinal cord sample was used to make cDNA. Each spinal cord sample was converted to cDNA using a Bio-Rad iScript RT supermix (Berkley, CA). Equivalent volumes of cDNA were used for PCR with degenerate primers targeting the VP1 gene (20) (PCR protocol: 95°C 3 min, 40x cycles of: 95°C 10 sec, 53°C 30 sec, 72°C 30 sec, melt curves: 65–95°C in 0.5°C steps). Samples were compared to a plasmid standard curve [11]. Temperature melt curves temperature and slope were used to assess the quality of each sample. The starting genome copy number for each dilution of the standard curve was estimated using spectrophotometer data. The starting genome copy number per μL was correlated to the resulting cycle threshold (Ct) value at each dilution in the standard curve, and the standard curve equation was calculated using Excel (Microsoft, Redmond, WA).

### Immunohistochemistry

Mice were anaesthetized with isoflurane before perfusion. Mice were perfused intracardially with 4% Na-periodate-lysine-paraformaldehyde fixative (PLP, 0.01M sodium periodate, 0.075M lysine, 2.0% paraformaldehyde, 0.037M phosphate) before tissue removal. Spinal cords were post-fixed in PLP at 4°C overnight and then transferred to a solution of 30% sucrose in PBS (pH 7.4) until saturated. Spinal cords were then kept in a 1:1 mixture of a 20% sucrose solution in PBS and Optimal Cutting Temperature (O.C.T.) compound for 1–2 days at 4°C before being placed into embedding cups filled with O.C.T. compound and rapidly frozen. Ten μm thick serial frozen sections were cut on a cryostat. Sections were then incubated with primary antibodies overnight: rabbit anti-ChAT (1:100; #ab178850, Abcam, Cambridge, UK), rabbit anti-EV-D68 VP2 (1:100; #GTX132314, GeneTex, Irvine, CA), and mouse anti-Neun (1:50; #MAB377, Millipore, Billerica, MA). Alexa Fluor goat-anti-rabbit 488 or goat-anti-mouse 546 (1:1000; Molecular Probes, Eugene, OR) were used for secondary antibody labeling. Nuclei were labeled with Hoechst 33342 (10 ng/mL; Invitrogen, Carlsbad, CA). Sections imaged at 100X were taken as two separate images in a single plane and stitched with FIJI [33, 34]. The 200X and 600X images were imaged as Z-series and flatted as brightest point projections. All images were taken on an FV-1000 confocal microscope (Olympus, Center Valley, PA) and brightened linearly for publication with FIJI.

### Electron microscopy

Paralyzed mice were perfused intracardially with ice-cold 0.1 M phosphate buffer followed by Karnovsky’s fixative (2.5% glutaraldehyde, 2% paraformaldehyde, in 0.1 M phosphate buffer at pH 7.4) until rigid. Extracted spinal cords were post-fixed at 4°C overnight. Cervical and lumbar regions were dissected from the spinal cords and sent to the Electron Microscopy Core for further processing. Spinal cord regions were sliced on a vibratome into 200 μm-thick sections for resin embedding. Ultra-thin sections (65 nm) were cut on a Reichert Ultracut E from a small trapezoid positioned over the anterior horns and were picked up on Formvar-coated slot grids (EMS) and then stained with uranyl acetate and lead citrate. Sections were imaged on an FEI Technai G2 transmission electron microscope (Hillsboro, OR) with an AMT digital camera (Woburn, MA).

### Antibody neutralization assay

RD cells were grown to form a sub-confluent cell layer in a 96-well plate. Serum samples were heat inactivated for 30 minutes at 56°C. To perform the virus neutralization assay, stock virus (US/MO/14-18947) at a concentration of 100-TCID_50_ and serum dilutions starting at a 1:10 in eleven 2-fold steps were mixed and incubated for 1 hour at 37°C. Diluent and serum were mixed 1:1 and used as a control. After incubation, dilution mixtures were inoculated into separate wells of the cell plate, and the plate was placed in a 33°C 5% CO_2_ incubator. Cells in the plate were observed for evidence of CPE for 2 weeks. The reciprocal of the highest dilution that inhibited viral CPE was taken as the neutralizing titer. Sera that failed to protect at 1:10 were considered to have an undetectable titer that was graphed as zero.

### Metagenomic deep sequencing

See “Tissue Processing and Analysis” for information on anesthesia and tissue collection. Nucleic acid was extracted from 50 μL of spinal cords lysate with the Direct-zol RNA Kit (Zymo Research, Irvine, CA) or 50 μL virus culture using the automated Qiagen EZ1 robotic instrument (Qiagen, Valencia, CA), followed by treatment with Turbo DNase, then randomly reverse transcribed to cDNA with random hexamer primers. NGS libraries were constructed by using the Nextera XT kit (Illumina, San Diego, CA), followed by 161-bp, single-end sequencing on an Illumina MiSeq instrument. Metagenomic next-generation sequencing data were analyzed using the SURPI ("sequence-based ultrarapid pathogen identification") computational pipeline [27], which identifies viruses, bacteria, fungi, and parasites by computational subtraction of human host sequences followed by nucleotide and translated nucleotide (protein) alignment of remaining reads to all microbial sequences present in the National Center for Biotechnology Information (NCBI) GenBank database (as of December 2015). Raw SURPI outputs consisting of aligned microbial reads were taxonomically classified to the appropriate rank (family, genus, or species) by use of an in-house developed LCA (lowest common ancestor) algorithm incorporating the SNAP nucleotide aligner (v0.15.4) [35]. Summary read count tables were generated for viruses, bacteria, and eukaryotic organisms including fungi and parasites outside of the phylum Chordata or kingdom Viridiplantae ("non-chordate eukaryotes"). Reads aligning to enterovirus D68 (EV-D68) were automatically mapped using SURPI to the US/MO/14-18947 genome (GenBank accession KM851225.1) for determination of percent genomic coverage achieved.

### Statistics

Statistical analyses were done using GraphPad Prism ver 6 (Carlsbad, CA). Comparisons of categorical data were done with a Fisher’s exact test. Data were considered significant at *p* ≤ 0.05 for all statistical tests.

### Data deposition

Re-sequenced EV-D68 genomes corresponding to the culture stocks of the EV-D68 strains used for infection and recovered viral RNA from passaged spinal cord lysates have been deposited in the National Center for Biotechnology Information (NCBI) GenBank database under the following accession numbers (KU844178-KU844181). Deep sequencing data corresponding to viral culture supernatants and the spinal cord lysates used for EV-D68 genome assembly and metagenomic analyses have been deposited in the NCBI Sequence Read Archive (accession number SRP055445). The assembled genomes and associated deep sequencing data can also be found as part of NCBI BioProject PRJNA266569.

## Supporting information

S1 Movie A mouse pup at day post-infection (dpi) 3 with left forelimb paralysis following intracerebral inoculation with EV-D68 strain MO/14-18947. (MP4)

S2 Movie A mouse pup at day post-infection (dpi) 12 with persistent bilateral hindlimb paralysis following intracerebral inoculation with EV-D68 strain MO/14-18947. (MP4)

S3 Movie A mouse at day post-infection (dpi) 28 with persistent left forelimb and left hindlimb paralysis following intracerebral inoculation with EV-D68 strain MO/14-18947. (MP4)

S4 Movie A mouse pup at day post-infection (dpi) 3 with left hindlimb paralysis following intramuscular inoculation with EV-D68 strain MO/14-18947. (MP4)

S5 Movie A mouse pup at day post-infection (dpi) 12 with right forelimb paralysis following intranasal inoculation with EV-D68 strain MO/14-18947. (MP4)

S1 Fig Control spinal cord sections did not stain positive for EV-D68 VP2. 200X original magnification images from the cervical spinal cord of a mock-injected control mouse stained for EV-D68 VP2 (green), NeuN (magenta), and Hoechst 33341 (blue) at 4 days post-intracerebral injection of control media. Faint green background staining was occasionally seen in the neuropil, but not in the cell bodies. Images were collected and processed under conditions identical to those utilized in Fig 5C. The scale bar is 200 μm.
(TIF)

S2 Fig EV-D68 phylogeny based on VP1 sequence. Phylogeny of EV-D68 based on VP1 gene sequence from the prototypic ancestral Fermon and Rhyne strains to more recent members of clade B1, as well as clades A, B, and C. Alignment of the VP1 gene, the most diverse gene in the viral genome, has historically been the established method for genotyping enteroviruses [31]. By phylogenetic analysis, the homology of the VP1 region amongst members of the B1 clade is very high (>98% pairwise identity) versus 85–95% between other clades (A and C) [18]. In 2014, only EV-D68 strains from clade B1 were isolated from AFM patients examined in one study [18]. In the current study, strains from clades A, B, and B1 produced paralysis in neonatal mice (a 2014 clade C strain was not available for testing). Strains tested in this paper are bolded and indicated with a red dot (paralytogenic) or a black dot (non- or rarely paralytogenic). Black text indicates strains found in respiratory samples from patients with respiratory disease. Red text indicates strains found in respiratory samples from patients with AFM. The P00X numbers next to each strain indicated the number of passages of the strains in cultured cells since collection or since being received from the sample archive (BEI resources).
(TIF)

S1 Text Microbial identification by metagenomic next-generation sequencing. Metagenomic next-generation sequencing data were analyzed using the SURPI ("sequence-based ultrarapid pathogen identification") computational pipeline [27] which identifies viruses, bacteria, fungi, and parasites by computational subtraction of human host sequences followed by nucleotide and translated nucleotide (protein) alignment of remaining reads to all microbial sequences present in the National Center for Biotechnology Information (NCBI) GenBank database (as of December 2015). Raw SURPI outputs consisting of aligned microbial reads were taxonomically classified to the appropriate rank (family, genus, or species) by use of an in-house developed LCA (lowest common ancestor) algorithm incorporating the SNAP nucleotide aligner (v0.15.4) [35]. Summary read count tables were generated for viruses, bacteria, and eukaryotic organisms including fungi and parasites outside of the phylum Chordata or kingdom Viridiplantae ("non-chordate eukaryotes") (Tables A-D). Reads aligning to enterovirus D68 (EV-D68) were automatically mapped using SURPI to the MO/14-18947 genome (GenBank accession KM851225.1) for determination of percent genomic coverage achieved.
(PDF)

S1 Table Mice (n = 4–5) were inoculated by intramuscular injection (left hindlimb) with EV-D68 strains Fermon, Rhyne, CA/14-4231, or MO/14-18947. On day post-injection (dpi) 6, mice were sacrificed, and muscle tissue of the injected leg was harvested for TCID_50_ analysis. At this time point, all MO/14-18947 mice were showing signs of paralysis in the injected limb, while none of the Fermon, Rhyne, or CA/14-4231 mice had signs of paralysis. Viral infection was detected within the muscle from mice injected with MO/14-18947, CA/14-4231, and Rhyne as calculated by TCID_50_/mg of muscle tissue. Fermon was not detected in the muscle tissue of any mouse.
(PDF)

## References

[1] Schieble JH, Fox VL, Lennette EH. A probable new human picornavirus associated with respiratory diseases. Am J Epidemiol. 1967;85(2):297–310. 4960233 10.1093/oxfordjournals.aje.a120693

[2] Pons-Salort M, Parker EP, Grassly NC. The epidemiology of non-polio enteroviruses: recent advances and outstanding questions. Curr Opin Infect Dis. 2015;28(5):479–87. 10.1097/QCO.0000000000000187 26203854 PMC6624138

[3] Blomqvist S, Savolainen C, Raman L, Roivainen M, Hovi T. Human rhinovirus 87 and enterovirus 68 represent a unique serotype with rhinovirus and enterovirus features. J Clin Microbiol. 2002;40(11):4218–23. PubMed Central PMCID: PMC139630. 10.1128/JCM.40.11.4218-4223.2002 12409401 PMC139630

[4] Oberste MS, Maher K, Schnurr D, Flemister MR, Lovchik JC, Peters H, et al Enterovirus 68 is associated with respiratory illness and shares biological features with both the enteroviruses and the rhinoviruses. J Gen Virol. 2004;85(Pt 9):2577–84. 10.1099/vir.0.79925-0 15302951

[5] Khetsuriani N, Lamonte-Fowlkes A, Oberst S, Pallansch MA, Centers for Disease C, Prevention. Enterovirus surveillance—United States, 1970–2005. MMWR Surveill Summ. 2006;55(8):1–20. 16971890

[6] Holm-Hansen CC, Midgley SE, Fischer TK. Global emergence of enterovirus D68: a systematic review. Lancet Infect Dis. 2016. 10.1016/S1473-3099(15)00543-5 26929196

[7] Tokarz R, Firth C, Madhi SA, Howie SR, Wu W, Sall AA, et al Worldwide emergence of multiple clades of enterovirus 68. J Gen Virol. 2012;93(Pt 9):1952–8. PubMed Central PMCID: PMC3542132. 10.1099/vir.0.043935-0 22694903 PMC3542132

[8] Non-Polio Enterovirus | About EV-D68 | Enterovirus D68 | CDC (2016). Cdcgov. Available: https://www.cdc.gov/non-polio-enterovirus/about/ev-d68.html. Accessed 23 December 2016.

[9] Midgley CM, Watson JT, Nix WA, Curns AT, Rogers SL, Brown BA, et al Severe respiratory illness associated with a nationwide outbreak of enterovirus D68 in the USA (2014): a descriptive epidemiological investigation. Lancet Respir Med. 2015;3(11):879–87. 10.1016/S2213-2600(15)00335-5 26482320 PMC5693332

[10] Messacar K, Abzug MJ, Dominguez SR. 2014 outbreak of enterovirus D68 in North America. J Med Virol. 2016;88(5):739–45. 10.1002/jmv.24410 26489019

[11] Wylie TN, Wylie KM, Buller RS, Cannella M, Storch GA. Development and Evaluation of an Enterovirus D68 Real-Time Reverse Transcriptase PCR Assay. J Clin Microbiol. 2015;53(8):2641–7. PubMed Central PMCID: PMC4508392. 10.1128/JCM.00923-15 26063859 PMC4508392

[12] Leshem E (2015) Notes from the Field: Acute Flaccid Myelitis Among Persons Aged ≤21 Years—United States, August 1–November 13, 2014. Cdcgov. Available: https://www.cdc.gov/mmwr/preview/mmwrhtml/mm6353a3.htm. Accessed 23 December 2016. PMC4646045 25577990

[13] Messacar K, Schreiner TL, Maloney JA, Wallace A, Ludke J, Oberste MS, et al A cluster of acute flaccid paralysis and cranial nerve dysfunction temporally associated with an outbreak of enterovirus D68 in children in Colorado, USA. Lancet. 2015;385(9978):1662–71. 10.1016/S0140-6736(14)62457-0 25638662

[14] Nelson GR, Bonkowsky JL, Doll E, Green M, Hedlund GL, Moore KR, et al Recognition and Management of Acute Flaccid Myelitis in Children. Pediatr Neurol. 2016;55:17–21. 10.1016/j.pediatrneurol.2015.10.007 26621554

[15] Pastula DM, Aliabadi N, Haynes AK, Messacar K, Schreiner T, Maloney J, et al Acute neurologic illness of unknown etiology in children—Colorado, August-September 2014. MMWR Morb Mortal Wkly Rep. 2014;63(40):901–2. 25299607 PMC4584613

[16] Van Haren K, Ayscue P, Waubant E, Clayton A, Sheriff H, Yagi S, et al Acute Flaccid Myelitis of Unknown Etiology in California, 2012–2015. JAMA. 2015;314(24):2663–71. 10.1001/jama.2015.17275 26720027

[17] Acute Flaccid Myelitis | AFM Surveillance | CDC (2017). Cdcgov. Available: https://www.cdc.gov/acute-flaccid-myelitis/afm-surveillance.html. Accessed 9 February 2017.

[18] Greninger AL, Naccache SN, Messacar K, Clayton A, Yu G, Somasekar S, et al A novel outbreak enterovirus D68 strain associated with acute flaccid myelitis cases in the USA (2012–14): a retrospective cohort study. Lancet Infect Dis. 2015;15(6):671–82. 10.1016/S1473-3099(15)70093-9 25837569 PMC6027625

[19] Sejvar JJ, Lopez AS, Cortese MM, Leshem E, Pastula DM, Miller L, et al Acute Flaccid Myelitis in the United States, August-December 2014: Results of Nationwide Surveillance. Clin Infect Dis. 2016. 10.1093/cid/ciw372 PMC5709818 27318332

[20] Messacar K, Schreiner TL, Van Haren K, Yang M, Glaser CA, Tyler KL, et al Acute flaccid myelitis: A clinical review of US cases 2012–2015. Ann Neurol. 2016;80(3):326–38. 10.1002/ana.24730 27422805 PMC5098271

[21] Maloney JA, Mirsky DM, Messacar K, Dominguez SR, Schreiner T, Stence NV. MRI findings in children with acute flaccid paralysis and cranial nerve dysfunction occurring during the 2014 enterovirus D68 outbreak. AJNR Am J Neuroradiol. 2015;36(2):245–50. 10.3174/ajnr.A4188 25414005 PMC7965662

[22] Aliabadi N MK, Pastula DM, Robinson CC, Leshem E, Sejvar JJ, et al Enterovirus D68 infection in children with acute flaccid myelitis, Colorado, USA, 2014. Emerg Infect Dis. 2016. 10.3201/eid2208.151949 PMC4982171 27434186

[23] Kreuter JD, Barnes A, McCarthy JE, Schwartzman JD, Oberste MS, Rhodes CH, et al A fatal central nervous system enterovirus 68 infection. Arch Pathol Lab Med. 2011;135(6):793–6. 10.1043/2010-0174-CR.1 21631275

[24] Castelhano-Carlos MJ, Sousa N, Ohl F, Baumans V. Identification methods in newborn C57BL/6 mice: a developmental and behavioural evaluation. Lab Anim. 2010;44(2):88–103. 10.1258/la.2009.009044 19854756

[25] Non-Polio Enterovirus | EV-D68 Photos | Enterovirus D68 | CDC (2016). Cdcgov. Available: https://www.cdc.gov/non-polio-enterovirus/resources-ev68-photos.html. Accessed 23 December 2016.

[26] Kaufmann SH, Schaible UE. 100th anniversary of Robert Koch's Nobel Prize for the discovery of the tubercle bacillus. Trends Microbiol. 2005;13(10):469–75. 10.1016/j.tim.2005.08.003 16112578

[27] Naccache SN, Federman S, Veeraraghavan N, Zaharia M, Lee D, Samayoa E, et al A cloud-compatible bioinformatics pipeline for ultrarapid pathogen identification from next-generation sequencing of clinical samples. Genome Res. 2014;24(7):1180–92. PubMed Central PMCID: PMC4079973. 10.1101/gr.171934.113 24899342 PMC4079973

[28] Tyler KL, McPhee DA, Fields BN. Distinct pathways of viral spread in the host determined by reovirus S1 gene segment. Science. 1986;233(4765):770–4. 3016895 10.1126/science.3016895

[29] Gromeier M, Wimmer E. Mechanism of injury-provoked poliomyelitis. J Virol. 1998;72(6):5056–60. PubMed Central PMCID: PMC110068. 9573275 10.1128/jvi.72.6.5056-5060.1998 PMC110068

[30] Centers for Disease Control and Prevention. Clusters of acute respiratory illness associated with human enterovirus 68—Asia, Europe, and United States, 2008–2010. MMWR Morb Mortal Wkly Rep. 2011;60(38):1301–4. 21956405

[31] Oberste MS, Maher K, Kilpatrick DR, Pallansch MA. Molecular evolution of the human enteroviruses: correlation of serotype with VP1 sequence and application to picornavirus classification. J Virol. 1999;73(3):1941–8. PubMed Central PMCID: PMC104435. 9971773 10.1128/jvi.73.3.1941-1948.1999 PMC104435

[32] Quick ED, Leser JS, Clarke P, Tyler KL. Activation of intrinsic immune responses and microglial phagocytosis in an ex vivo spinal cord slice culture model of West Nile virus infection. J Virol. 2014;88(22):13005–14. PubMed Central PMCID: PMC4249089. 10.1128/JVI.01994-14 25165111 PMC4249089

[33] Preibisch S, Saalfeld S, Tomancak P. Globally optimal stitching of tiled 3D microscopic image acquisitions. Bioinformatics. 2009;25(11):1463–5. PubMed Central PMCID: PMC2682522. 10.1093/bioinformatics/btp184 19346324 PMC2682522

[34] Schindelin J, Arganda-Carreras I, Frise E, Kaynig V, Longair M, Pietzsch T, et al Fiji: an open-source platform for biological-image analysis. Nat Methods. 2012;9(7):676–82. PubMed Central PMCID: PMC3855844. 10.1038/nmeth.2019 22743772 PMC3855844

[35] Zaharia M, Bolosky, WJ, Curtis, K; Fox, A, Patterson, D, Shenker, S, Stoica, I, Karp, RM, Sittler, T. Faster and more accurate sequence alignment with SNAP. arXiv.org 1111.55722011.

