# Supplementary figures and images for: A mouse model of paralytic myelitis caused by enterovirus D68
Source: PLoS Pathog. 2017 Feb 23;13(2):e1006199. doi: 10.1371/journal.ppat.1006199 (PMC5322875; doi:10.1371/journal.ppat.1006199)

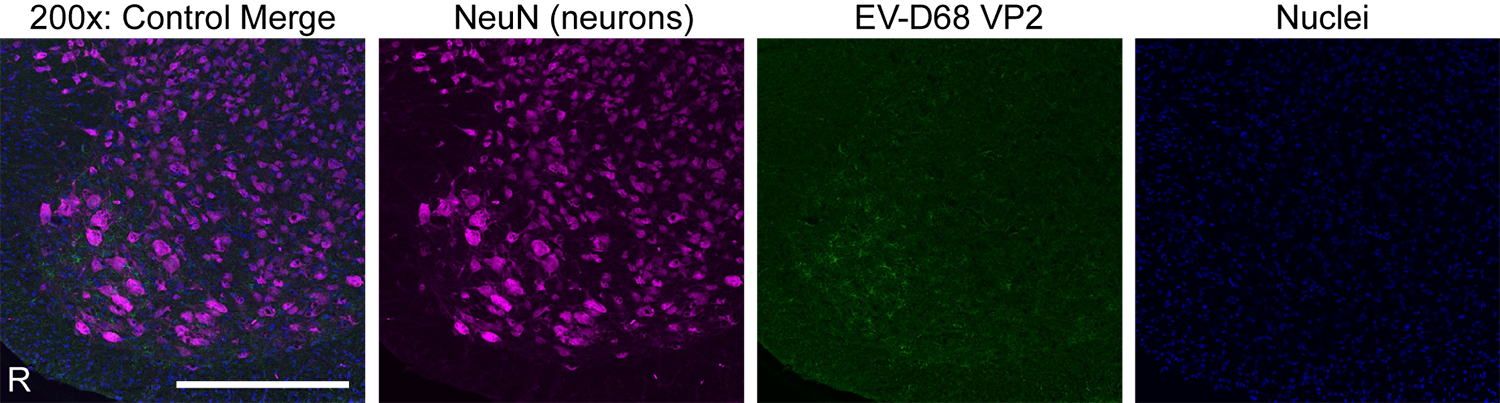

Supplement: S1 Fig — 200X original magnification images from the cervical spinal cord of a mock-injected control mouse stained for EV-D68 VP2 (green), NeuN (magenta), and Hoechst 33341 (blue) at 4 days post-intracerebral injection of control media. Faint green background staining was occasionally seen in the neuropil, but not in the cell bodies. Images were collected and processed under conditions identical to those utilized in Fig 5C. The scale bar is 200 μm. (TIF) [file ppat.1006199.s006.tif]

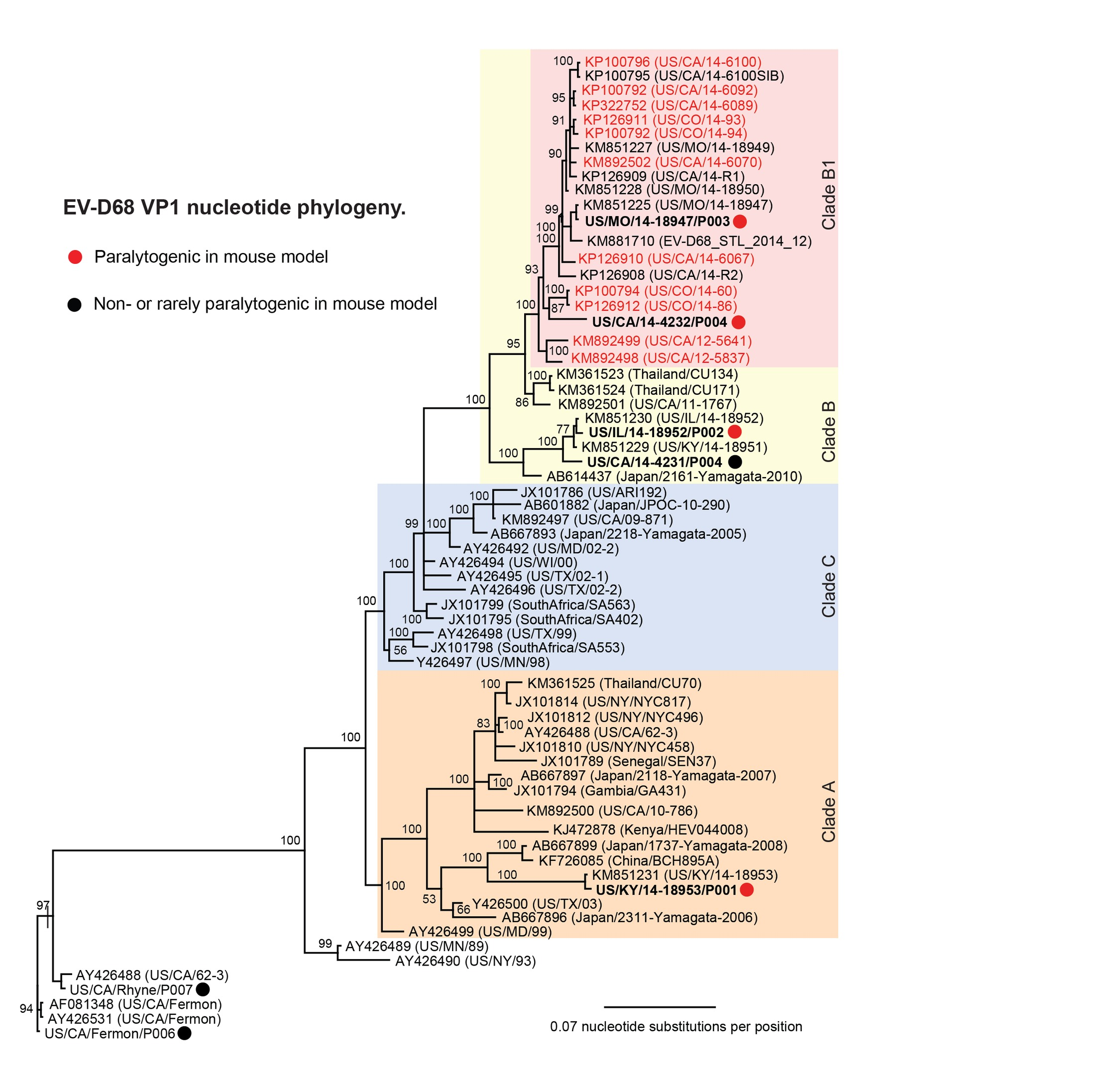

Supplement: S2 Fig — Phylogeny of EV-D68 based on VP1 gene sequence from the prototypic ancestral Fermon and Rhyne strains to more recent members of clade B1, as well as clades A, B, and C. Alignment of the VP1 gene, the most diverse gene in the viral genome, has historically been the established method for genotyping enteroviruses [31]. By phylogenetic analysis, the homology of the VP1 region amongst members of the B1 clade is very high (>98% pairwise identity) versus 85–95% between other clades (A and C) [18]. In 2014, only EV-D68 strains from clade B1 were isolated from AFM patients examined in one study [18]. In the current study, strains from clades A, B, and B1 produced paralysis in neonatal mice (a 2014 clade C strain was not available for testing). Strains tested in this paper are bolded and indicated with a red dot (paralytogenic) or a black dot (non- or rarely paralytogenic). Black text indicates strains found in respiratory samples from patients with respiratory disease. Red text indicates strains found in respiratory samples from patients with AFM. The P00X numbers next to each strain indicated the number of passages of the strains in cultured cells since collection or since being received from the sample archive (BEI resources). (TIF) [file ppat.1006199.s007.tif]
