# Supplementary material for: A mouse model of paralytic myelitis caused by enterovirus D68
Source: PLoS Pathog. 2017 Feb 23;13(2):e1006199. doi: 10.1371/journal.ppat.1006199 (PMC5322875; doi:10.1371/journal.ppat.1006199)
Supplement: S1 Text — Metagenomic next-generation sequencing data were analyzed using the SURPI ("sequence-based ultrarapid pathogen identification") computational pipeline [27] which identifies viruses, bacteria, fungi, and parasites by computational subtraction of human host sequences followed by nucleotide and translated nucleotide (protein) alignment of remaining reads to all microbial sequences present in the National Center for Biotechnology Information (NCBI) GenBank database (as of December 2015). Raw SURPI outputs consisting of aligned microbial reads were taxonomically classified to the appropriate rank (family, genus, or species) by use of an in-house developed LCA (lowest common ancestor) algorithm incorporating the SNAP nucleotide aligner (v0.15.4) [35]. Summary read count tables were generated for viruses, bacteria, and eukaryotic organisms including fungi and parasites outside of the phylum Chordata or kingdom Viridiplantae ("non-chordate eukaryotes") (Tables A-D). Reads aligning to enterovirus D68 (EV-D68) were automatically mapped using SURPI to the MO/14-18947 genome (GenBank accession KM851225.1) for determination of percent genomic coverage achieved. (PDF) [file ppat.1006199.s008.pdf]

S1 Text.

Microbial identification by metagenomic next-generation sequencing.

Metagenomic next-generation sequencing data were analyzed using the SURPI ("sequence-based ultrarapid pathogen identification") computational pipeline [27] which identifies viruses, bacteria, fungi, and parasites by computational subtraction of human host sequences followed by nucleotide and translated nucleotide (protein) alignment of remaining reads to all microbial sequences present in the National Center for Biotechnology Information (NCBI) GenBank database (as of December 2015). Raw SURPI outputs consisting of aligned microbial reads were taxonomically classified to the appropriate rank (family, genus, or species) by use of an in-house developed LCA (lowest common ancestor) algorithm incorporating the SNAP nucleotide aligner (v0.15.4) [35]. Summary read count tables were generated for viruses, bacteria, and eukaryotic organisms including fungi and parasites outside of the phylum *Chordata* or kingdom *Viridiplantae* ("non-chordate eukaryotes") (Tables A-D). Reads aligning to enterovirus D68 (EV-D68) were automatically mapped using SURPI to the MO/14-18947 genome (GenBank accession KM851225.1) for determination of percent genomic coverage achieved.

27. Naccache SN, Federman S, Veeraraghavan N, Zaharia M, Lee D, Samayoa E, et al. A cloud-compatible bioinformatics pipeline for ultrarapid pathogen identification from next-generation sequencing of clinical samples. Genome Res. 2014;24(7):1180-92. doi: 10.1101/gr.171934.113. PubMed PMID: 24899342; PubMed Central PMCID: PMC4079973.

35. Zaharia M, Bolosky, WJ, Curtis, K; Fox, A, Patterson, D, Shenker, S, Stoica, I, Karp, RM, Sittler, T. Faster and more accurate sequence alignment with SNAP. arXiv.org 1111.55722011.

Table A: Enteroviral reads by SURPI for each step in the Koch's Postulate experiment.

	# of raw reads	# of EV-D68 reads	% of EV-D68 reads	% of genome coverage
Koch's Postulate Step 1: US/MO/14-18947 stock	1,603,835	938759	58.53214327	99.99
**Koch's Postulate Step 2: SC lysate from original dpi 4 mouse injected with MO/14-18947**	2,184,354	226	0.010346308	84.19
Koch's Postulate Step 3: RD cell lysate inoculated with SC from the mouse in Step 2	1,176,226	19839	1.686665658	98.31
**Koch's Postulate Step 4: SC lysate from dpi 3 animal injected with RD cell lysate from Step 3**	1,952,028	2657	0.136114851	97.44
**Koch's Postulate Step 4: SC lysate from dpi 3 animal injected with RD cell lysate from Step 3**	2,239,917	3687	0.164604313	99.48
**Koch's Postulate Step 4: SC lysate from dpi 3 animal injected with RD cell lysate from Step 3**	2,303,907	4902	0.212769005	99.52

Table B. Viral Reads Detected by SURPI for the Koch's Postulate SC lysates.

Species	Genus	Family	Tag	Koch'sPostulate Step 2: SC lysate (dpi 4)	Koch's Postulate Step 4: SC lysate (dpi 3)	Koch's Postulate Step 4: SC lysate (dpi 4)	Koch's Postulate Step 4: SC lysate (dpi 5)
*	Enterovirus	Picornaviridae	host-01|human|vertebrates;	52	477	580	850
Enterovirus B	Enterovirus	Picornaviridae	host-01|human|vertebrates;	0	0	2	0
Enterovirus C	Enterovirus	Picornaviridae	host-01|human|vertebrates;	0	0	0	2
Enterovirus D	Enterovirus	Picornaviridae	host-01|human|vertebrates;	174	2,195	3,114	4,055
Enterovirus sp.	Enterovirus	Picornaviridae	host-01|human|vertebrates;	0	3	2	2
Human enterovirus	Enterovirus	Picornaviridae	host-01|human|vertebrates;	0	3	0	0
STL polyomavirus	Polyomavirus	Polyomaviridae	host-01|human|vertebrates;	0	0	0	0
AKT8 retrovirus		Retroviridae	host-01|human|vertebrates;	0	0	0	1
Gallid herpesvirus 2	Mardivirus	Herpesviridae	host-02|vertebrates;	0	0	0	1
*	Gammaretrovirus	Retroviridae	host-02|vertebrates;	0	0	0	0
Murine leukemia virus	Gammaretrovirus	Retroviridae	host-02|vertebrates;	3	0	0	0
Mus musculus mobilized endo	Gammaretrovirus	Retroviridae	host-02|vertebrates;	5	0	1	0
Escherichia phage rv5		Myoviridae	host-19|bacteria;	1	0	0	0
Streptococcus phage EJ-1		Myoviridae	host-19|bacteria;	0	0	0	0
*	T7likevirus	Podoviridae	host-19|bacteria;	0	0	1	0
*		Siphoviridae	host-19|bacteria;	5	0	0	0
Salmonella phage Vi II-E1		Siphoviridae	host-19|bacteria;	0	0	0	0
Streptococcus phage Abc2		Siphoviridae	host-19|bacteria;	4	0	0	0
*	*	*		24	12	31	40
Tipula oleracea nudivirus		Nudiviridae		0	0	0	0
unidentified phage				2	0	0	0

Table C. Bacterial Reads Detected by SURPI for the Koch's Postulate SC lysates.

Species	Genus	Family	Koch'sPostulate Step 2: SC lysate (dpi 4)	Koch's Postulate Step 4: SC lysate (dpi 3)	Koch's Postulate Step 4: SC lysate (dpi 4)	Koch's Postulate Step 4: SC lysate (dpi 5)
*	*	*	340	93	88	61
*	*	Acetobacteraceae	0	0	0	0
*	Acidiphilium	Acetobacteraceae	0	0	0	0
Acidiphilium multivorum	Acidiphilium	Acetobacteraceae	0	0	0	0
Gluconacetobacter diazotrophicus	Gluconacetobacter	Acetobacteraceae	3	0	0	0
Komagataeibacter xylinus	Komagataeibacter	Acetobacteraceae	0	0	2	0
Oleomonas sagaranensis	Oleomonas	Acetobacteraceae	1	0	0	0
*	Acidaminococcus	Acidaminococcaceae	0	0	0	0
Acidaminococcus fermentans	Acidaminococcus	Acidaminococcaceae	0	0	0	1
Acidaminococcus intestini	Acidaminococcus	Acidaminococcaceae	0	0	0	0
Acidimicrobium ferrooxidans	Acidimicrobium	Acidimicrobiaceae	0	0	0	0
Ilumatobacter coccineus	Ilumatobacter	Acidimicrobiaceae	2	0	1	0
Candidatus Koribacter versatilis	Candidatus Koribacter	Acidobacteriaceae	3	0	0	0
Granulicella mallensis	Granulicella	Acidobacteriaceae	0	0	0	0
Terriglobus saanensis	Terriglobus	Acidobacteriaceae	0	0	0	0
Acidothermus cellulolyticus	Acidothermus	Acidothermaceae	0	0	0	0
Actinobaculum schaalii	Actinobaculum	Actinomycetaceae	0	0	0	0
Arcanobacterium haemolyticum	Arcanobacterium	Actinomycetaceae	0	1	0	0
Mobiluncus curtisii	Mobiluncus	Actinomycetaceae	0	0	0	0
Trueperella pyogenes	Trueperella	Actinomycetaceae	0	0	0	0
Aerococcus urinae	Aerococcus	Aerococcaceae	2	0	0	0
*	Aeromonas	Aeromonadaceae	0	1	0	0
Aeromonas hydrophila	Aeromonas	Aeromonadaceae	1	0	0	0
Aeromonas media	Aeromonas	Aeromonadaceae	0	2	1	0
Aeromonas salmonicida	Aeromonas	Aeromonadaceae	2	0	0	0
Oceanimonas sp. GK1	Oceanimonas	Aeromonadaceae	0	0	0	0
*	*	Alcaligenaceae	0	0	0	0
Achromobacter xylosoxidans	Achromobacter	Alcaligenaceae	2	0	1	1
Advenella mimigardefordensis	Advenella	Alcaligenaceae	0	0	0	0
*	Bordetella	Alcaligenaceae	2	0	0	0
Bordetella bronchiseptica	Bordetella	Alcaligenaceae	0	0	0	0
Bordetella holmesii	Bordetella	Alcaligenaceae	0	0	1	0
Bordetella petrii	Bordetella	Alcaligenaceae	0	0	0	0
Castellaniella defragrans	Castellaniella	Alcaligenaceae	0	0	0	0
Alcanivorax pacificus	Alcanivorax	Alcanivoracaceae	2	0	0	0
Alteromonas macleodii	Alteromonas	Alteromonadaceae	1	0	0	0
Glaciecola psychrophila	Glaciecola	Alteromonadaceae	0	0	0	0
Marinobacter similis	Marinobacter	Alteromonadaceae	0	0	0	0
Anaerolinea thermophila	Anaerolinea	Anaerolineaceae	0	0	0	0
*	Anaeromyxobacter	Anaeromyxobacterace	0	0	1	0
Anaeromyxobacter dehalogenans	Anaeromyxobacter	Anaeromyxobacterace	2	0	1	0
Anaeromyxobacter sp. Fw109-5	Anaeromyxobacter	Anaeromyxobacterace	0	0	0	0
Anaeromyxobacter sp. K	Anaeromyxobacter	Anaeromyxobacterace	0	0	0	0
*	Wolbachia	Anaplasmataceae	1	0	0	0
Amphibacillus xylanus	Amphibacillus	Bacillaceae	0	0	0	0
*	Bacillus	Bacillaceae	0	0	0	1
Bacillus amyloliquefaciens	Bacillus	Bacillaceae	2	0	0	0
Bacillus firmus	Bacillus	Bacillaceae	0	0	0	0
Bacillus megaterium	Bacillus	Bacillaceae	0	0	3	0
Bacillus sp. OxB-1	Bacillus	Bacillaceae	1	0	0	0
*	Geobacillus	Bacillaceae	0	0	0	0
Geobacillus stearothermophilus	Geobacillus	Bacillaceae	0	0	0	0
Geobacillus thermodenitrificans	Geobacillus	Bacillaceae	0	0	0	0
Lysinibacillus sp. 13S34_air	Lysinibacillus	Bacillaceae	2	0	1	0
Lysinibacillus varians	Lysinibacillus	Bacillaceae	0	0	0	0
*	Bacteroides	Bacteroidaceae	5	8	1	0
Bacteroides coprosuis	Bacteroides	Bacteroidaceae	0	0	1	0
Bacteroides dorei	Bacteroides	Bacteroidaceae	2	0	1	0
Bacteroides fragilis	Bacteroides	Bacteroidaceae	25	12	13	3
Bacteroides helcogenes	Bacteroides	Bacteroidaceae	2	0	1	1
Bacteroides ovatus	Bacteroides	Bacteroidaceae	5	0	0	0
Bacteroides salanitronis	Bacteroides	Bacteroidaceae	1	3	0	0
Bacteroides thetaiotaomicron	Bacteroides	Bacteroidaceae	0	2	3	1
Bacteroides vulgatus	Bacteroides	Bacteroidaceae	0	3	1	1
Bacteroides xylanisolvens	Bacteroides	Bacteroidaceae	6	1	1	4
*	Bartonella	Bartonellaceae	0	0	0	0
Bartonella bacilliformis	Bartonella	Bartonellaceae	0	0	0	0
Beutenbergia cavernae	Beutenbergia	Beutenbergiaceae	9	0	2	1
*	Bifidobacterium	Bifidobacteriaceae	6	2	0	0
Bifidobacterium adolescentis	Bifidobacterium	Bifidobacteriaceae	0	0	0	0
Bifidobacterium animalis	Bifidobacterium	Bifidobacteriaceae	1	0	0	0
Bifidobacterium asteroides	Bifidobacterium	Bifidobacteriaceae	0	0	0	0
Bifidobacterium bifidum	Bifidobacterium	Bifidobacteriaceae	4	0	1	0
Bifidobacterium breve	Bifidobacterium	Bifidobacteriaceae	0	0	0	0
Bifidobacterium longum	Bifidobacterium	Bifidobacteriaceae	2	0	0	0
Bifidobacterium pseudolongum	Bifidobacterium	Bifidobacteriaceae	10	3	1	0
Bifidobacterium scardovii	Bifidobacterium	Bifidobacteriaceae	1	0	0	0
Bifidobacterium thermophilum	Bifidobacterium	Bifidobacteriaceae	0	3	0	2
Gardnerella vaginalis	Gardnerella	Bifidobacteriaceae	0	0	0	0

*	*	Bradyrhizobiaceae	0	1	0	0
*	Bradyrhizobium	Bradyrhizobiaceae	1	0	0	0
Bradyrhizobium diazoefficiens	Bradyrhizobium	Bradyrhizobiaceae	2	0	0	0
Bradyrhizobium japonicum	Bradyrhizobium	Bradyrhizobiaceae	0	0	2	0
Bradyrhizobium oligotrophicum	Bradyrhizobium	Bradyrhizobiaceae	1	0	0	0
Bradyrhizobium sp. BTAi1	Bradyrhizobium	Bradyrhizobiaceae	0	0	0	0
Bradyrhizobium sp. ORS 278	Bradyrhizobium	Bradyrhizobiaceae	0	0	0	0
Bradyrhizobium sp. S23321	Bradyrhizobium	Bradyrhizobiaceae	4	0	0	0
Oligotropha carboxidovorans	Oligotropha	Bradyrhizobiaceae	0	2	0	0
Rhodopseudomonas palustris	Rhodopseudomonas	Bradyrhizobiaceae	10	0	0	1
*	*	Brucellaceae	0	0	0	0
Ochrobactrum anthropi	Ochrobactrum	Brucellaceae	1	0	0	0
*	*	Burkholderiaceae	7	0	0	0
*	Burkholderia	Burkholderiaceae	5	5	6	1
Burkholderia ambifaria	Burkholderia	Burkholderiaceae	1	1	0	0
Burkholderia cenocepacia	Burkholderia	Burkholderiaceae	0	0	0	0
Burkholderia cepacia	Burkholderia	Burkholderiaceae	0	1	0	0
Burkholderia contaminans	Burkholderia	Burkholderiaceae	0	0	0	0
Burkholderia gladioli	Burkholderia	Burkholderiaceae	0	0	0	0
Burkholderia glumae	Burkholderia	Burkholderiaceae	3	0	0	0
Burkholderia lata	Burkholderia	Burkholderiaceae	5	0	3	0
Burkholderia multivorans	Burkholderia	Burkholderiaceae	5	0	0	0
Burkholderia oklahomensis	Burkholderia	Burkholderiaceae	1	0	0	0
Burkholderia phymatum	Burkholderia	Burkholderiaceae	3	0	0	0
Burkholderia pseudomallei	Burkholderia	Burkholderiaceae	0	0	0	0
Burkholderia rhizoxinica	Burkholderia	Burkholderiaceae	2	0	0	0
Burkholderia sp. CCGE1003	Burkholderia	Burkholderiaceae	1	0	0	0
Burkholderia sp. RPE64	Burkholderia	Burkholderiaceae	0	0	1	0
*	Cupriavidus	Burkholderiaceae	0	0	0	1
Cupriavidus basilensis	Cupriavidus	Burkholderiaceae	0	0	0	0
Cupriavidus metallidurans	Cupriavidus	Burkholderiaceae	0	0	0	0
Cupriavidus necator	Cupriavidus	Burkholderiaceae	4	1	0	0
Cupriavidus pinatubonensis	Cupriavidus	Burkholderiaceae	3	1	0	0
Cupriavidus taiwanensis	Cupriavidus	Burkholderiaceae	4	0	0	0
*	Ralstonia	Burkholderiaceae	0	0	0	0
Ralstonia pickettii	Ralstonia	Burkholderiaceae	1	1	1	0
Ralstonia solanacearum	Ralstonia	Burkholderiaceae	2	0	1	0
Ralstonia syzygii	Ralstonia	Burkholderiaceae	0	1	0	0
blood disease bacterium R229	Ralstonia	Burkholderiaceae	0	0	0	0
Arcobacter butzleri	Arcobacter	Campylobacteraceae	0	0	0	0
*	Campylobacter	Campylobacteraceae	0	0	1	0
Campylobacter concisus	Campylobacter	Campylobacteraceae	4	0	0	0
Campylobacter curvus	Campylobacter	Campylobacteraceae	0	0	1	0
Campylobacter fetus	Campylobacter	Campylobacteraceae	6	0	0	0
Campylobacter insulaenigrae	Campylobacter	Campylobacteraceae	0	0	3	0
Campylobacter jejuni	Campylobacter	Campylobacteraceae	0	0	0	0
Campylobacter lari	Campylobacter	Campylobacteraceae	0	0	0	0
Campylobacter peloridis	Campylobacter	Campylobacteraceae	0	0	0	0
*	Carnobacterium	Carnobacteriaceae	3	0	0	2
Carnobacterium maltaromaticum	Carnobacterium	Carnobacteriaceae	0	0	0	0
Carnobacterium sp. 17-4	Carnobacterium	Carnobacteriaceae	2	2	0	7
Carnobacterium sp. WN1359	Carnobacterium	Carnobacteriaceae	74	40	47	54
Granulicatella adiacens	Granulicatella	Carnobacteriaceae	2	0	0	0
Catenulispora acidiphila	Catenulispora	Catenulisporaceae	3	0	0	0
*	*	Caulobacteraceae	1	0	1	1
Brevundimonas subvibrioides	Brevundimonas	Caulobacteraceae	21	5	3	2
*	Caulobacter	Caulobacteraceae	2	0	0	0
Caulobacter segnis	Caulobacter	Caulobacteraceae	2	0	0	0
Caulobacter sp. K31	Caulobacter	Caulobacteraceae	3	0	2	0
Caulobacter vibrioides	Caulobacter	Caulobacteraceae	1	0	1	0
Phenylobacterium zucineum	Phenylobacterium	Caulobacteraceae	1	2	0	0
*	Cellulomonas	Cellulomonadaceae	1	0	1	0
Cellulomonas fimi	Cellulomonas	Cellulomonadaceae	15	1	0	1
Cellulomonas flavigena	Cellulomonas	Cellulomonadaceae	8	2	0	2
[Cellvibrio] gilvus	Cellulomonas	Cellulomonadaceae	14	1	2	0
Oerskovia turbata	Oerskovia	Cellulomonadaceae	0	0	0	0
Niabella soli	Niabella	Chitinophagaceae	0	0	1	0
Chlamydia muridarum	Chlamydia	Chlamydiaceae	6	2	0	0
Chlamydia trachomatis	Chlamydia	Chlamydiaceae	12	0	0	0
Chlorobium limicola	Chlorobium	Chlorobiaceae	0	0	0	0
Prosthecochloris aestuarii	Prosthecochloris	Chlorobiaceae	0	0	0	0
*	*	Chromatiaceae	0	0	0	0
Allochromatium vinosum	Allochromatium	Chromatiaceae	0	0	0	0
Marichromatium purpuratum	Marichromatium	Chromatiaceae	0	0	0	0
Chromobacterium violaceum	Chromobacterium	Chromobacteriaceae	0	0	2	0
*	Clostridium	Clostridiaceae	2	1	0	0
Clostridium baratii	Clostridium	Clostridiaceae	0	3	0	0
Clostridium beijerinckii	Clostridium	Clostridiaceae	0	0	0	0
Clostridium botulinum	Clostridium	Clostridiaceae	0	0	1	4
Clostridium pasteurianum	Clostridium	Clostridiaceae	0	0	2	0
Clostridium perfringens	Clostridium	Clostridiaceae	2	0	1	0
Clostridium saccharobutylicum	Clostridium	Clostridiaceae	1	0	0	0

Clostridium saccharoperbutylacetonic	Clostridium	Clostridiaceae	0	2	0	0
Clostridium sp. SY8519	Clostridium	Clostridiaceae	1	0	0	0
Clostridium sp. enrichment culture clo	Clostridium	Clostridiaceae	1	0	0	0
Thermaerobacter marianensis	Thermaerobacter	Clostridiales Family XV	0	0	0	0
Colwellia psychrerythraea	Colwellia	Colwelliaceae	0	0	0	0
*	*	Comamonadaceae	6	0	2	0
*	Acidovorax	Comamonadaceae	9	0	0	0
Acidovorax avenae	Acidovorax	Comamonadaceae	2	0	0	0
Acidovorax citrulli	Acidovorax	Comamonadaceae	1	0	0	0
Acidovorax ebreus	Acidovorax	Comamonadaceae	0	0	0	0
Acidovorax sp. JS42	Acidovorax	Comamonadaceae	4	0	1	0
Acidovorax sp. KKS102	Acidovorax	Comamonadaceae	10	2	0	3
Acidovorax sp. TP4	Acidovorax	Comamonadaceae	1	0	0	0
Alicycliphilus denitrificans	Alicycliphilus	Comamonadaceae	3	0	0	0
Comamonas testosteroni	Comamonas	Comamonadaceae	0	3	0	0
*	Delftia	Comamonadaceae	0	1	3	1
Delftia acidovorans	Delftia	Comamonadaceae	1	0	0	0
Delftia sp. Cs1-4	Delftia	Comamonadaceae	0	0	2	0
Polaromonas naphthalenivorans	Polaromonas	Comamonadaceae	3	0	0	2
Polaromonas sp. JS666	Polaromonas	Comamonadaceae	2	1	0	0
Ramlibacter tataouinensis	Ramlibacter	Comamonadaceae	2	0	2	1
Rhodoferax ferrireducens	Rhodoferax	Comamonadaceae	0	0	1	0
Variovorax paradoxus	Variovorax	Comamonadaceae	13	6	4	0
Verminephrobacter eiseniae	Verminephrobacter	Comamonadaceae	0	1	1	0
Comamonadaceae bacterium A1		Comamonadaceae	0	1	0	0
Comamonadaceae bacterium B1		Comamonadaceae	2	0	0	0
Conexibacter woesei	Conexibacter	Conexibacteraceae	1	1	1	0
*	*	Coriobacteriaceae	1	0	0	0
Adlercreutzia equolifaciens	Adlercreutzia	Coriobacteriaceae	0	0	0	1
Coriobacterium glomerans	Coriobacterium	Coriobacteriaceae	0	0	0	0
Gordonibacter pamelaeae	Gordonibacter	Coriobacteriaceae	0	0	0	0
Olsenella uli	Olsenella	Coriobacteriaceae	0	0	0	0
Slackia heliotrinireducens	Slackia	Coriobacteriaceae	1	0	0	0
Coriobacteriaceae bacterium 68-1-3		Coriobacteriaceae	4	0	1	0
*	Corynebacterium	Corynebacteriaceae	30	3	12	4
Corynebacterium ammoniagenes	Corynebacterium	Corynebacteriaceae	1	1	0	0
Corynebacterium argentoratense	Corynebacterium	Corynebacteriaceae	0	0	0	0
Corynebacterium aurimucosum	Corynebacterium	Corynebacteriaceae	3	0	0	0
Corynebacterium callunae	Corynebacterium	Corynebacteriaceae	34	17	2	2
Corynebacterium casei	Corynebacterium	Corynebacteriaceae	13	4	13	9
Corynebacterium diphtheriae	Corynebacterium	Corynebacteriaceae	3	0	0	0
Corynebacterium doosanense	Corynebacterium	Corynebacteriaceae	6	7	3	2
Corynebacterium efficiens	Corynebacterium	Corynebacteriaceae	13	6	15	6
Corynebacterium falsenii	Corynebacterium	Corynebacteriaceae	0	2	0	3
Corynebacterium glutamicum	Corynebacterium	Corynebacteriaceae	42	17	22	21
Corynebacterium glycinophilum	Corynebacterium	Corynebacteriaceae	0	2	2	0
Corynebacterium halotolerans	Corynebacterium	Corynebacteriaceae	2	1	1	2
Corynebacterium humireducens	Corynebacterium	Corynebacteriaceae	14	3	7	4
Corynebacterium imitans	Corynebacterium	Corynebacteriaceae	1	0	0	1
Corynebacterium jeikeium	Corynebacterium	Corynebacteriaceae	2	1	0	0
Corynebacterium kroppenstedtii	Corynebacterium	Corynebacteriaceae	1	0	0	0
Corynebacterium marinum	Corynebacterium	Corynebacteriaceae	15	2	5	4
Corynebacterium maris	Corynebacterium	Corynebacteriaceae	7	6	4	7
Corynebacterium resistens	Corynebacterium	Corynebacteriaceae	3	4	0	0
Corynebacterium singulare	Corynebacterium	Corynebacteriaceae	5	0	0	2
Corynebacterium sp. ATCC 6931	Corynebacterium	Corynebacteriaceae	5	3	3	1
Corynebacterium sp. L2-79-05	Corynebacterium	Corynebacteriaceae	0	0	0	0
Corynebacterium terpenotabidum	Corynebacterium	Corynebacteriaceae	1	2	0	0
Corynebacterium urealyticum	Corynebacterium	Corynebacteriaceae	0	0	1	1
Corynebacterium ureicelerivorans	Corynebacterium	Corynebacteriaceae	17	1	2	0
Corynebacterium variabile	Corynebacterium	Corynebacteriaceae	3	4	7	4
Corynebacterium vitaeruminis	Corynebacterium	Corynebacteriaceae	2	1	3	1
Corynebacterium xerosis	Corynebacterium	Corynebacteriaceae	1	0	0	0
Owenweeksia hongkongensis	Owenweeksia	Cryomorphaceae	3	0	0	0
Echinicola vietnamensis	Echinicola	Cyclobacteriaceae	1	0	0	0
Stigmatella aurantiaca	Stigmatella	Cystobacteraceae	0	0	0	0
Dyadobacter fermentans	Dyadobacter	Cytophagaceae	0	0	0	0
Fibrella aestuarina	Fibrella	Cytophagaceae	1	0	0	0
*	Hymenobacter	Cytophagaceae	7	1	0	0
Hymenobacter sp. APR13	Hymenobacter	Cytophagaceae	15	4	0	1
Hymenobacter sp. DG25B	Hymenobacter	Cytophagaceae	3	0	1	3
Hymenobacter swuensis	Hymenobacter	Cytophagaceae	15	8	0	2
Leadbetterella byssophila	Leadbetterella	Cytophagaceae	0	0	0	0
Runella slithyformis	Runella	Cytophagaceae	0	0	0	0
*	Deinococcus	Deinococcaceae	0	0	1	0
Deinococcus deserti	Deinococcus	Deinococcaceae	0	0	0	0
Deinococcus geothermalis	Deinococcus	Deinococcaceae	9	0	2	1
Deinococcus gobiensis	Deinococcus	Deinococcaceae	7	2	2	1
Deinococcus maricopensis	Deinococcus	Deinococcaceae	4	0	0	1
Deinococcus peraridilitoris	Deinococcus	Deinococcaceae	4	1	0	0
Deinococcus proteolyticus	Deinococcus	Deinococcaceae	2	1	0	0
Deinococcus radiodurans	Deinococcus	Deinococcaceae	5	3	1	2

Deinococcus swuensis	Deinococcus	Deinococcaceae	17	7	5	3
Brachybacterium faecium	Brachybacterium	Dermabacteraceae	56	8	9	7
*	*	Dermacoccaceae	0	0	0	0
Dermacoccus nishinomiyaensis	Dermacoccus	Dermacoccaceae	13	4	5	3
Kytococcus sedentarius	Kytococcus	Dermacoccaceae	5	8	8	1
Desulfarculus baarsii	Desulfarculus	Desulfarculaceae	1	0	0	0
Desulfotalea psychrophila	Desulfotalea	Desulfobulbaceae	0	0	0	0
Desulfomicrobium baculatum	Desulfomicrobium	Desulfomicrobiaceae	2	0	1	0
Desulfovibrio desulfuricans	Desulfovibrio	Desulfovibrionaceae	0	0	0	0
Dietzia sp. D5	Dietzia	Dietziaceae	2	0	0	0
Dietzia sp. DQ12-45-1b	Dietzia	Dietziaceae	1	0	0	0
Thioalkalivibrio sp. K90mix	Thioalkalivibrio	Ectothiorhodospiracea	0	2	0	0
Thioalkalivibrio thiocyanoxidans	Thioalkalivibrio	Ectothiorhodospiracea	1	0	0	0
*	*	Enterobacteriaceae	132	27	14	7
Buchnera aphidicola	Buchnera	Enterobacteriaceae	0	0	0	0
Cedecea neteri	Cedecea	Enterobacteriaceae	0	0	0	0
*	Citrobacter	Enterobacteriaceae	0	0	0	4
Citrobacter freundii	Citrobacter	Enterobacteriaceae	0	3	0	0
Citrobacter koseri	Citrobacter	Enterobacteriaceae	0	0	0	0
Cronobacter turicensis	Cronobacter	Enterobacteriaceae	0	2	0	0
Dickeya dadantii	Dickeya	Enterobacteriaceae	0	0	0	0
*	Enterobacter	Enterobacteriaceae	0	0	0	0
Enterobacter asburiae	Enterobacter	Enterobacteriaceae	3	0	0	0
Enterobacter cloacae	Enterobacter	Enterobacteriaceae	1	0	1	0
Erwinia billingiae	Erwinia	Enterobacteriaceae	0	0	2	0
Erwinia sp. Ejp617	Erwinia	Enterobacteriaceae	0	0	0	0
*	Escherichia	Enterobacteriaceae	0	0	0	0
Escherichia coli	Escherichia	Enterobacteriaceae	56	27	9	2
*	Klebsiella	Enterobacteriaceae	0	0	0	2
Klebsiella oxytoca	Klebsiella	Enterobacteriaceae	0	0	0	0
Klebsiella pneumoniae	Klebsiella	Enterobacteriaceae	0	0	0	0
*	Pantoea	Enterobacteriaceae	0	0	1	0
Pantoea ananatis	Pantoea	Enterobacteriaceae	1	0	0	0
Pantoea sp. PSNIH1	Pantoea	Enterobacteriaceae	71	21	23	24
Pantoea sp. PSNIH2	Pantoea	Enterobacteriaceae	1	0	0	0
Pantoea vagans	Pantoea	Enterobacteriaceae	1	0	1	0
*	Pectobacterium	Enterobacteriaceae	0	0	0	0
Pectobacterium carotovorum	Pectobacterium	Enterobacteriaceae	0	0	1	0
Providencia stuartii	Providencia	Enterobacteriaceae	6	0	0	0
Rahnella aquatilis	Rahnella	Enterobacteriaceae	0	0	0	0
Raoultella ornithinolytica	Raoultella	Enterobacteriaceae	1	0	0	0
Salmonella enterica	Salmonella	Enterobacteriaceae	0	0	0	0
*	Serratia	Enterobacteriaceae	3	0	0	0
Serratia marcescens	Serratia	Enterobacteriaceae	0	0	0	1
Serratia plymuthica	Serratia	Enterobacteriaceae	2	0	0	0
Serratia proteamaculans	Serratia	Enterobacteriaceae	0	0	1	0
Shigella dysenteriae	Shigella	Enterobacteriaceae	1	0	0	0
Shigella sonnei	Shigella	Enterobacteriaceae	3	0	0	0
Sodalis sp. HS1	Sodalis	Enterobacteriaceae	1	0	0	0
Xenorhabdus nematophila	Xenorhabdus	Enterobacteriaceae	0	0	0	0
*	Yersinia	Enterobacteriaceae	0	0	0	0
*	Enterococcus	Enterococcaceae	7	0	4	3
Enterococcus casseliflavus	Enterococcus	Enterococcaceae	1	0	0	0
Enterococcus faecalis	Enterococcus	Enterococcaceae	0	3	0	0
Enterococcus faecium	Enterococcus	Enterococcaceae	3	5	0	0
Enterococcus hirae	Enterococcus	Enterococcaceae	0	0	0	0
Enterococcus mundtii	Enterococcus	Enterococcaceae	0	1	0	0
Enterococcus pseudoavium	Enterococcus	Enterococcaceae	0	0	0	0
Tetragenococcus halophilus	Tetragenococcus	Enterococcaceae	3	0	0	0
Faecalitalea cylindroides	Faecalitalea	Erysipelotrichaceae	6	0	0	0
Erythrobacter litoralis	Erythrobacter	Erythrobacteraceae	0	0	0	2
Eubacterium limosum	Eubacterium	Eubacteriaceae	1	3	0	0
Eubacterium rectale	Eubacterium	Eubacteriaceae	12	3	0	0
[Eubacterium] eligens	Eubacterium	Eubacteriaceae	7	0	3	0
*	*	Flavobacteriaceae	1	0	0	0
Aequorivita sublithincola	Aequorivita	Flavobacteriaceae	0	0	0	0
Capnocytophaga ochracea	Capnocytophaga	Flavobacteriaceae	0	0	0	0
Cellulophaga algicola	Cellulophaga	Flavobacteriaceae	0	0	0	0
Chryseobacterium haifense	Chryseobacterium	Flavobacteriaceae	0	0	2	0
Chryseobacterium sp. StRB126	Chryseobacterium	Flavobacteriaceae	3	0	0	0
Elizabethkingia anophelis	Elizabethkingia	Flavobacteriaceae	1	0	2	0
*	Flavobacterium	Flavobacteriaceae	0	0	0	0
Flavobacterium branchiophilum	Flavobacterium	Flavobacteriaceae	0	0	0	2
Flavobacterium frigidimaris	Flavobacterium	Flavobacteriaceae	0	0	0	0
Flavobacterium indicum	Flavobacterium	Flavobacteriaceae	0	0	0	1
Flavobacterium johnsoniae	Flavobacterium	Flavobacteriaceae	2	3	0	4
Flavobacterium psychrophilum	Flavobacterium	Flavobacteriaceae	0	0	0	0
Formosa agariphila	Formosa	Flavobacteriaceae	2	0	0	0
Maribacter sp. HTCC2170	Maribacter	Flavobacteriaceae	0	0	0	0
Muricauda ruestringensis	Muricauda	Flavobacteriaceae	0	0	0	0
Myroides profundi	Myroides	Flavobacteriaceae	0	0	0	3
Ornithobacterium rhinotracheale	Ornithobacterium	Flavobacteriaceae	0	1	0	0

Psychroflexus torquis	Psychroflexus	Flavobacteriaceae	0	0	0	0
Riemerella anatipestifer	Riemerella	Flavobacteriaceae	0	2	0	0
Robiginitalea biformata	Robiginitalea	Flavobacteriaceae	0	0	1	0
Weeksella virosa	Weeksella	Flavobacteriaceae	0	0	1	2
Zobellia galactanivorans	Zobellia	Flavobacteriaceae	0	1	1	0
Flavobacteriaceae bacterium 3519-10		Flavobacteriaceae	0	1	2	2
Flavobacteriaceae bacterium JJC		Flavobacteriaceae	0	0	0	0
Francisella philomiragia	Francisella	Francisellaceae	0	0	0	0
*	Frankia	Frankiaceae	2	0	0	0
Frankia alni	Frankia	Frankiaceae	0	0	0	0
Frankia sp. CN3	Frankia	Frankiaceae	0	0	0	0
Frankia sp. CcI3	Frankia	Frankiaceae	1	0	0	0
Frankia sp. EAN1pec	Frankia	Frankiaceae	4	0	0	0
Frankia sp. EuI1c	Frankia	Frankiaceae	2	0	1	2
Frankia symbiont of Datisca glomerat	Frankia	Frankiaceae	1	0	0	2
Fusobacterium nucleatum	Fusobacterium	Fusobacteriaceae	0	2	0	0
Sideroxydans lithotrophicus	Sideroxydans	Gallionellaceae	0	0	0	0
Geobacter sp. M21	Geobacter	Geobacteraceae	0	0	0	0
*	*	Geodermatophilaceae	4	0	0	0
Blastococcus saxobsidens	Blastococcus	Geodermatophilaceae	5	1	1	0
Geodermatophilus obscurus	Geodermatophilus	Geodermatophilaceae	5	0	3	1
Modestobacter marinus	Modestobacter	Geodermatophilaceae	6	0	3	3
Stackebrandtia nassauensis	Stackebrandtia	Glycomycetaceae	2	0	1	1
Gordonia bronchialis	Gordonia	Gordoniaceae	0	2	0	0
Gordonia cholesterolivorans	Gordonia	Gordoniaceae	0	0	1	0
Gordonia polyisoprenivorans	Gordonia	Gordoniaceae	0	0	0	0
Gordonia sp. KTR9	Gordonia	Gordoniaceae	0	0	0	0
Helicobacter cinaedi	Helicobacter	Helicobacteraceae	0	0	1	0
Wolinella succinogenes	Wolinella	Helicobacteraceae	0	0	0	0
Hyphomicrobium denitrificans	Hyphomicrobium	Hyphomicrobiaceae	1	0	0	0
Pelagibacterium halotolerans	Pelagibacterium	Hyphomicrobiaceae	1	1	0	0
Rhodomicrobium vannielii	Rhodomicrobium	Hyphomicrobiaceae	0	0	0	1
Hyphomonas neptunium	Hyphomonas	Hyphomonadaceae	0	0	0	0
Idiomarina loihiensis	Idiomarina	Idiomarinaceae	0	0	0	0
*	*	Intrasporangiaceae	0	0	0	0
Intrasporangium calvum	Intrasporangium	Intrasporangiaceae	15	5	7	1
Serinicoccus profundi	Serinicoccus	Intrasporangiaceae	0	2	0	0
Terrabacter sp. DBF63	Terrabacter	Intrasporangiaceae	1	0	0	0
Jonesia denitrificans	Jonesia	Jonesiaceae	0	0	2	0
Kineococcus radiotolerans	Kineococcus	Kineosporiaceae	6	1	1	1
Haliangium ochraceum	Haliangium	Kofleriaceae	2	0	0	0
*	*	Lachnospiraceae	1	1	3	0
*	Blautia	Lachnospiraceae	0	1	0	0
[Ruminococcus] obeum	Blautia	Lachnospiraceae	14	4	2	7
[Ruminococcus] torques	Blautia	Lachnospiraceae	3	0	2	0
Butyrivibrio fibrisolvens	Butyrivibrio	Lachnospiraceae	0	3	0	4
Butyrivibrio proteoclasticus	Butyrivibrio	Lachnospiraceae	0	0	0	2
Cellulosilyticum lentocellum	Cellulosilyticum	Lachnospiraceae	0	0	1	0
Coprococcus catus	Coprococcus	Lachnospiraceae	27	6	8	1
Coprococcus sp. ART55/1	Coprococcus	Lachnospiraceae	8	0	0	1
Lachnoclostridium phytofermentans	Lachnoclostridium	Lachnospiraceae	1	0	0	3
[Clostridium] saccharolyticum	Lachnoclostridium	Lachnospiraceae	14	0	2	1
*	Roseburia	Lachnospiraceae	0	0	0	0
Roseburia hominis	Roseburia	Lachnospiraceae	11	5	1	0
Roseburia intestinalis	Roseburia	Lachnospiraceae	4	7	1	1
Lachnospiraceae bacterium 14-2		Lachnospiraceae	0	0	0	0
*	Lactobacillus	Lactobacillaceae	72	10	30	36
Lactobacillus acidophilus	Lactobacillus	Lactobacillaceae	3	1	4	2
Lactobacillus amylovorus	Lactobacillus	Lactobacillaceae	19	6	6	7
Lactobacillus brevis	Lactobacillus	Lactobacillaceae	0	0	1	0
Lactobacillus crispatus	Lactobacillus	Lactobacillaceae	2	0	2	0
Lactobacillus delbrueckii	Lactobacillus	Lactobacillaceae	4	2	3	0
Lactobacillus fermentum	Lactobacillus	Lactobacillaceae	1	0	0	0
Lactobacillus helveticus	Lactobacillus	Lactobacillaceae	9	2	4	2
Lactobacillus johnsonii	Lactobacillus	Lactobacillaceae	25	4	2	2
Lactobacillus kefiranofaciens	Lactobacillus	Lactobacillaceae	3	0	0	0
Lactobacillus mucosae	Lactobacillus	Lactobacillaceae	0	1	0	0
Lactobacillus pentosus	Lactobacillus	Lactobacillaceae	0	0	0	0
Lactobacillus reuteri	Lactobacillus	Lactobacillaceae	46	16	22	19
Lactobacillus ruminis	Lactobacillus	Lactobacillaceae	5	2	0	2
Lactobacillus sakei	Lactobacillus	Lactobacillaceae	0	0	0	0
Lactobacillus sanfranciscensis	Lactobacillus	Lactobacillaceae	0	0	0	0
Lactobacillus sp. wkB8	Lactobacillus	Lactobacillaceae	0	0	0	0
Pediococcus pentosaceus	Pediococcus	Lactobacillaceae	0	1	0	0
Leptotrichia buccalis	Leptotrichia	Leptotrichiaceae	0	0	0	0
Leuconostoc carnosum	Leuconostoc	Leuconostocaceae	0	0	0	0
Leuconostoc citreum	Leuconostoc	Leuconostocaceae	0	0	0	2
Leuconostoc gelidum	Leuconostoc	Leuconostocaceae	0	0	0	1
Leuconostoc mesenteroides	Leuconostoc	Leuconostocaceae	2	0	0	0
Leuconostoc pseudomesenteroides	Leuconostoc	Leuconostocaceae	0	0	0	0
Weissella ceti	Weissella	Leuconostocaceae	7	0	0	0
Weissella paramesenteroides	Weissella	Leuconostocaceae	0	3	0	0

Weissella thailandensis	Weissella	Leuconostocaceae	15	4	2	3
Listeria ivanovii	Listeria	Listeriaceae	0	0	0	0
Melioribacter roseus	Melioribacter	Melioribacteraceae	0	0	0	2
*	Methylobacterium	Methylobacteriaceae	6	0	0	0
Methylobacterium aquaticum	Methylobacterium	Methylobacteriaceae	1	1	0	0
Methylobacterium extorquens	Methylobacterium	Methylobacteriaceae	4	4	0	0
Methylobacterium nodulans	Methylobacterium	Methylobacteriaceae	0	0	0	0
Methylobacterium oryzae	Methylobacterium	Methylobacteriaceae	3	0	0	0
Methylobacterium populi	Methylobacterium	Methylobacteriaceae	6	0	0	0
Methylobacterium radiotolerans	Methylobacterium	Methylobacteriaceae	0	0	2	1
Methylobacterium sp. 4-46	Methylobacterium	Methylobacteriaceae	0	4	0	1
Methylococcus capsulatus	Methylococcus	Methylococcaceae	0	0	0	0
Methylocystis sp. SC2	Methylocystis	Methylocystaceae	0	0	0	0
*	*	Microbacteriaceae	7	0	0	0
Clavibacter michiganensis	Clavibacter	Microbacteriaceae	27	11	2	1
Leifsonia xyli	Leifsonia	Microbacteriaceae	18	3	7	1
*	Microbacterium	Microbacteriaceae	0	1	0	0
Microbacterium arborescens	Microbacterium	Microbacteriaceae	1	0	0	3
Microbacterium foliorum	Microbacterium	Microbacteriaceae	0	0	0	0
Microbacterium liquefaciens	Microbacterium	Microbacteriaceae	0	0	0	0
Microbacterium testaceum	Microbacterium	Microbacteriaceae	27	2	9	8
Rathayibacter toxicus	Rathayibacter	Microbacteriaceae	1	0	0	0
*	*	Micrococcaceae	3	1	0	1
*	Arthrobacter	Micrococcaceae	2	0	0	0
Arthrobacter arilaitensis	Arthrobacter	Micrococcaceae	18	2	5	0
Arthrobacter aurescens	Arthrobacter	Micrococcaceae	0	2	0	0
Arthrobacter chlorophenolicus	Arthrobacter	Micrococcaceae	3	0	0	0
Arthrobacter phenanthrenivorans	Arthrobacter	Micrococcaceae	5	1	5	1
Arthrobacter sp. 11W110_air	Arthrobacter	Micrococcaceae	7	7	4	2
Arthrobacter sp. FB24	Arthrobacter	Micrococcaceae	1	0	1	0
Arthrobacter sp. PAMC25486	Arthrobacter	Micrococcaceae	2	0	1	0
Arthrobacter sp. Rue61a	Arthrobacter	Micrococcaceae	0	0	0	0
Kocuria rhizophila	Kocuria	Micrococcaceae	20	2	3	5
Micrococcus luteus	Micrococcus	Micrococcaceae	26	6	16	4
Rothia dentocariosa	Rothia	Micrococcaceae	1	0	2	0
Rothia mucilaginosa	Rothia	Micrococcaceae	12	2	0	1
psychrophilic marine bacterium PS32		Micrococcaceae	3	0	0	0
*	*	Micromonosporaceae	1	0	0	0
*	Actinoplanes	Micromonosporaceae	0	0	0	0
Actinoplanes friuliensis	Actinoplanes	Micromonosporaceae	1	2	0	0
Actinoplanes missouriensis	Actinoplanes	Micromonosporaceae	1	0	0	0
Actinoplanes sp. N902-109	Actinoplanes	Micromonosporaceae	5	2	0	0
Actinoplanes sp. SE50/110	Actinoplanes	Micromonosporaceae	1	3	0	0
*	Micromonospora	Micromonosporaceae	9	0	0	4
Micromonospora sp. L5	Micromonospora	Micromonosporaceae	0	0	0	0
Micromonospora sp. TP-A0468	Micromonospora	Micromonosporaceae	0	0	0	0
*	Salinispora	Micromonosporaceae	0	0	0	0
Salinispora arenicola	Salinispora	Micromonosporaceae	2	0	0	0
Salinispora tropica	Salinispora	Micromonosporaceae	0	0	0	3
Verrucosispora maris	Verrucosispora	Micromonosporaceae	0	0	0	2
*	*	Moraxellaceae	0	0	0	0
*	Acinetobacter	Moraxellaceae	12	3	2	3
Acinetobacter baumannii	Acinetobacter	Moraxellaceae	8	4	5	5
Acinetobacter calcoaceticus	Acinetobacter	Moraxellaceae	1	1	0	1
Acinetobacter guillouiae	Acinetobacter	Moraxellaceae	8	0	2	7
Acinetobacter johnsonii	Acinetobacter	Moraxellaceae	0	2	0	0
Acinetobacter nosocomialis	Acinetobacter	Moraxellaceae	0	0	0	0
Acinetobacter oleivorans	Acinetobacter	Moraxellaceae	3	0	1	1
Acinetobacter radioresistens	Acinetobacter	Moraxellaceae	0	0	0	0
Acinetobacter sp.	Acinetobacter	Moraxellaceae	0	0	0	0
Acinetobacter sp. 26B2	Acinetobacter	Moraxellaceae	0	0	0	1
Acinetobacter sp. 35(2010)	Acinetobacter	Moraxellaceae	0	0	0	0
Acinetobacter sp. ADP1	Acinetobacter	Moraxellaceae	3	0	3	0
Acinetobacter sp. LUH5605	Acinetobacter	Moraxellaceae	0	0	0	0
Acinetobacter sp. M-1	Acinetobacter	Moraxellaceae	0	0	0	0
Acinetobacter sp. M131	Acinetobacter	Moraxellaceae	0	0	0	0
Acinetobacter sp. NFM2	Acinetobacter	Moraxellaceae	0	0	0	0
Moraxella catarrhalis	Moraxella	Moraxellaceae	1	1	2	1
Moraxella osloensis	Moraxella	Moraxellaceae	2	0	0	0
*	Psychrobacter	Moraxellaceae	2	2	0	1
Psychrobacter arcticus	Psychrobacter	Moraxellaceae	0	0	1	2
Psychrobacter cryohalolentis	Psychrobacter	Moraxellaceae	0	0	2	1
Psychrobacter maritimus	Psychrobacter	Moraxellaceae	3	1	0	0
Psychrobacter sp. G	Psychrobacter	Moraxellaceae	0	1	0	1
Psychrobacter sp. PRwf-1	Psychrobacter	Moraxellaceae	0	0	1	8
Moritella viscosa	Moritella	Moritellaceae	0	0	0	0
*	Mycobacterium	Mycobacteriaceae	25	0	2	3
Mycobacterium abscessus	Mycobacterium	Mycobacteriaceae	1	3	0	0
Mycobacterium arupense	Mycobacterium	Mycobacteriaceae	6	0	0	0
Mycobacterium avium	Mycobacterium	Mycobacteriaceae	9	5	0	0
Mycobacterium chubuense	Mycobacterium	Mycobacteriaceae	2	0	2	0
Mycobacterium gilvum	Mycobacterium	Mycobacteriaceae	4	3	3	1

Mycobacterium intracellulare	Mycobacterium	Mycobacteriaceae	1	0	0	0
Mycobacterium kansasii	Mycobacterium	Mycobacteriaceae	0	0	0	0
Mycobacterium marinum	Mycobacterium	Mycobacteriaceae	3	0	0	0
Mycobacterium neoaurum	Mycobacterium	Mycobacteriaceae	3	2	0	0
Mycobacterium rhodesiae	Mycobacterium	Mycobacteriaceae	3	0	0	0
Mycobacterium smegmatis	Mycobacterium	Mycobacteriaceae	5	0	0	0
Mycobacterium sp. CH-1	Mycobacterium	Mycobacteriaceae	0	0	0	4
Mycobacterium sp. JDM601	Mycobacterium	Mycobacteriaceae	0	0	0	2
Mycobacterium sp. JLS	Mycobacterium	Mycobacteriaceae	4	0	0	0
Mycobacterium sp. VKM Ac-1817D	Mycobacterium	Mycobacteriaceae	5	0	0	0
Mycobacterium tuberculosis	Mycobacterium	Mycobacteriaceae	1	0	0	0
Mycobacterium vanbaalenii	Mycobacterium	Mycobacteriaceae	0	0	0	0
Mycobacterium yongonense	Mycobacterium	Mycobacteriaceae	0	0	0	0
Mycoplasma agalactiae	Mycoplasma	Mycoplasmataceae	0	0	0	0
Mycoplasma cynos	Mycoplasma	Mycoplasmataceae	0	0	0	0
Corallococcus coralloides	Corallococcus	Myxococcaceae	1	0	0	0
Myxococcus fulvus	Myxococcus	Myxococcaceae	0	0	0	1
Myxococcus stipitatus	Myxococcus	Myxococcaceae	0	0	1	0
Myxococcus xanthus	Myxococcus	Myxococcaceae	2	0	0	0
Nakamurella multipartita	Nakamurella	Nakamurellaceae	7	5	0	0
Neisseria elongata	Neisseria	Neisseriaceae	0	2	0	0
Neisseria gonorrhoeae	Neisseria	Neisseriaceae	0	0	0	0
Neisseria lactamica	Neisseria	Neisseriaceae	0	0	0	0
Neisseria meningitidis	Neisseria	Neisseriaceae	0	0	0	1
Nocardia brasiliensis	Nocardia	Nocardiaceae	0	0	1	0
Nocardia cyriacigeorgica	Nocardia	Nocardiaceae	3	4	0	0
Nocardia farcinica	Nocardia	Nocardiaceae	0	2	0	2
Nocardia nova	Nocardia	Nocardiaceae	5	0	0	0
*	Rhodococcus	Nocardiaceae	2	0	0	0
Rhodococcus equi	Rhodococcus	Nocardiaceae	2	3	1	0
Rhodococcus erythropolis	Rhodococcus	Nocardiaceae	1	0	0	0
Rhodococcus fascians	Rhodococcus	Nocardiaceae	0	1	0	0
Rhodococcus jostii	Rhodococcus	Nocardiaceae	0	2	0	0
Rhodococcus opacus	Rhodococcus	Nocardiaceae	4	1	0	0
Rhodococcus pyridinivorans	Rhodococcus	Nocardiaceae	2	0	0	3
*	*	Nocardioidaceae	1	2	2	5
Kribbella flavida	Kribbella	Nocardioidaceae	2	1	1	0
Nocardioides sp. JS614	Nocardioides	Nocardioidaceae	15	5	12	1
Pimelobacter simplex	Pimelobacter	Nocardioidaceae	24	4	10	5
*	Nocardiopsis	Nocardiopsaceae	0	0	0	0
Nocardiopsis alba	Nocardiopsis	Nocardiopsaceae	3	0	0	0
Nocardiopsis dassonvillei	Nocardiopsis	Nocardiopsaceae	1	0	1	1
Thermobifida fusca	Thermobifida	Nocardiopsaceae	0	0	0	0
*	*	Nostocaceae	0	0	0	0
Anabaena cylindrica	Anabaena	Nostocaceae	0	0	3	2
Anabaena sp. 90	Anabaena	Nostocaceae	0	0	0	0
Cylindrospermum stagnale	Cylindrospermum	Nostocaceae	0	4	0	0
Nodularia spumigena	Nodularia	Nostocaceae	1	0	1	0
*	Nostoc	Nostocaceae	0	2	0	0
Nostoc punctiforme	Nostoc	Nostocaceae	0	0	0	3
Nostoc sp. PCC 7120	Nostoc	Nostocaceae	0	0	0	0
Nostoc sp. PCC 7524	Nostoc	Nostocaceae	0	0	1	0
Trichormus azollae	Trichormus	Nostocaceae	0	0	0	0
Oscillibacter valericigenes	Oscillibacter	Oscillospiraceae	17	1	2	0
*	*	Oxalobacteraceae	0	0	2	0
*	Collimonas	Oxalobacteraceae	1	0	0	0
Collimonas arenae	Collimonas	Oxalobacteraceae	2	0	0	1
Collimonas fungivorans	Collimonas	Oxalobacteraceae	8	0	5	0
Herbaspirillum seropedicae	Herbaspirillum	Oxalobacteraceae	8	2	1	4
Herminiimonas arsenicoxydans	Herminiimonas	Oxalobacteraceae	2	0	0	0
Janthinobacterium agaricidamnosum	Janthinobacterium	Oxalobacteraceae	9	8	0	2
Janthinobacterium lividum	Janthinobacterium	Oxalobacteraceae	0	0	0	0
Janthinobacterium sp. J3	Janthinobacterium	Oxalobacteraceae	3	0	0	0
Janthinobacterium sp. Marseille	Janthinobacterium	Oxalobacteraceae	1	0	1	0
*	Paenibacillus	Paenibacillaceae	0	0	0	0
Paenibacillus sabinae	Paenibacillus	Paenibacillaceae	0	0	0	0
Paenibacillus sp. FSL R7-0331	Paenibacillus	Paenibacillaceae	0	0	0	0
*	*	Pasteurellaceae	1	0	0	1
*	Actinobacillus	Pasteurellaceae	0	0	2	0
Actinobacillus equuli	Actinobacillus	Pasteurellaceae	4	0	0	0
Actinobacillus pleuropneumoniae	Actinobacillus	Pasteurellaceae	0	0	0	0
Actinobacillus succinogenes	Actinobacillus	Pasteurellaceae	0	2	0	2
Actinobacillus suis	Actinobacillus	Pasteurellaceae	0	0	0	0
Aggregatibacter actinomycetemcomit	Aggregatibacter	Pasteurellaceae	0	0	0	0
Aggregatibacter aphrophilus	Aggregatibacter	Pasteurellaceae	0	0	0	0
[Mannheimia] succiniciproducens	Basfia	Pasteurellaceae	1	0	2	0
Bibersteinia trehalosi	Bibersteinia	Pasteurellaceae	1	0	0	0
Gallibacterium anatis	Gallibacterium	Pasteurellaceae	11	7	6	0
Haemophilus influenzae	Haemophilus	Pasteurellaceae	0	0	1	3
Haemophilus parainfluenzae	Haemophilus	Pasteurellaceae	3	0	0	0
Haemophilus parasuis	Haemophilus	Pasteurellaceae	10	3	0	13
Histophilus somni	Histophilus	Pasteurellaceae	0	0	0	0

Mannheimia haemolytica	Mannheimia	Pasteurellaceae	0	0	0	0
Mannheimia varigena	Mannheimia	Pasteurellaceae	1	0	0	0
Pasteurella multocida	Pasteurella	Pasteurellaceae	2	0	0	0
Pelobacter propionicus	Pelobacter	Pelobacteraceae	0	0	0	0
Dehalobacter restrictus	Dehalobacter	Peptococcaceae	0	0	0	0
*	Desulfitobacterium	Peptococcaceae	0	0	0	0
Desulfitobacterium metallireducens	Desulfitobacterium	Peptococcaceae	0	0	0	0
Desulfotomaculum gibsoniae	Desulfotomaculum	Peptococcaceae	0	0	0	0
Filifactor alocis	Filifactor	Peptostreptococcacea	0	0	0	0
Peptoclostridium difficile	Peptoclostridium	Peptostreptococcacea	4	1	2	0
[Clostridium] sordellii	Peptoclostridium	Peptostreptococcacea	4	0	0	2
[Clostridium] sticklandii	Peptoclostridium	Peptostreptococcacea	2	1	0	0
Chelativorans sp. BNC1	Chelativorans	Phyllobacteriaceae	2	0	0	0
Mesorhizobium australicum	Mesorhizobium	Phyllobacteriaceae	2	1	0	0
Mesorhizobium ciceri	Mesorhizobium	Phyllobacteriaceae	3	0	3	0
Mesorhizobium huakuii	Mesorhizobium	Phyllobacteriaceae	0	0	0	2
Mesorhizobium loti	Mesorhizobium	Phyllobacteriaceae	0	3	0	0
Mesorhizobium opportunistum	Mesorhizobium	Phyllobacteriaceae	1	0	0	0
Isosphaera pallida	Isosphaera	Planctomycetaceae	0	1	0	0
Pirellula staleyi	Pirellula	Planctomycetaceae	3	0	0	0
Singulisphaera acidiphila	Singulisphaera	Planctomycetaceae	0	0	0	2
Kurthia gibsonii	Kurthia	Planococcaceae	0	0	0	0
Planococcus sp. PAMC 21323	Planococcus	Planococcaceae	1	0	6	0
Solibacillus silvestris	Solibacillus	Planococcaceae	6	1	0	0
Sorangium cellulosum	Sorangium	Polyangiaceae	3	0	1	0
Barnesiella viscericola	Barnesiella	Porphyromonadaceae	1	1	0	0
Odoribacter splanchnicus	Odoribacter	Porphyromonadaceae	6	2	0	0
Paludibacter propionicigenes	Paludibacter	Porphyromonadaceae	0	0	0	0
Parabacteroides distasonis	Parabacteroides	Porphyromonadaceae	0	0	0	0
Porphyromonas asaccharolytica	Porphyromonas	Porphyromonadaceae	0	0	0	0
Porphyromonas gingivalis	Porphyromonas	Porphyromonadaceae	1	0	0	0
Tannerella forsythia	Tannerella	Porphyromonadaceae	0	0	0	0
*	Prevotella	Prevotellaceae	3	0	0	0
Prevotella bryantii	Prevotella	Prevotellaceae	1	0	0	0
Prevotella dentalis	Prevotella	Prevotellaceae	10	0	2	0
Prevotella denticola	Prevotella	Prevotellaceae	4	0	0	0
Prevotella intermedia	Prevotella	Prevotellaceae	8	0	0	0
Prevotella melaninogenica	Prevotella	Prevotellaceae	7	3	0	0
Prevotella ruminicola	Prevotella	Prevotellaceae	12	1	2	0
*	*	Promicromonosporace	0	0	0	0
Isoptericola variabilis	Isoptericola	Promicromonosporace	14	1	2	2
Xylanimonas cellulosilytica	Xylanimonas	Promicromonosporace	4	4	0	1
Microlunatus phosphovorus	Microlunatus	Propionibacteriaceae	5	4	4	0
*	Propionibacterium	Propionibacteriaceae	0	1	1	0
Propionibacterium acidipropionici	Propionibacterium	Propionibacteriaceae	10	1	2	1
Propionibacterium acnes	Propionibacterium	Propionibacteriaceae	302	90	82	89
Propionibacterium avidum	Propionibacterium	Propionibacteriaceae	1	0	0	1
Propionibacterium freudenreichii	Propionibacterium	Propionibacteriaceae	2	0	3	0
Propionibacterium propionicum	Propionibacterium	Propionibacteriaceae	1	4	3	0
Pseudoalteromonas atlantica	Pseudoalteromonas	Pseudoalteromonadac	0	0	0	0
Pseudoalteromonas haloplanktis	Pseudoalteromonas	Pseudoalteromonadac	0	0	0	0
Pseudoalteromonas sp. A28	Pseudoalteromonas	Pseudoalteromonadac	0	0	0	0
Pseudoalteromonas sp. OCN003	Pseudoalteromonas	Pseudoalteromonadac	0	0	0	0
*	*	Pseudomonadaceae	1	0	0	0
Azotobacter chroococcum	Azotobacter	Pseudomonadaceae	0	1	0	0
Azotobacter vinelandii	Azotobacter	Pseudomonadaceae	0	0	0	0
Cellvibrio japonicus	Cellvibrio	Pseudomonadaceae	2	0	0	0
*	Pseudomonas	Pseudomonadaceae	14	0	4	10
Pseudomonas aeruginosa	Pseudomonas	Pseudomonadaceae	2	3	0	0
Pseudomonas alkylphenolia	Pseudomonas	Pseudomonadaceae	0	0	0	0
Pseudomonas anguilliseptica	Pseudomonas	Pseudomonadaceae	0	0	0	0
Pseudomonas balearica	Pseudomonas	Pseudomonadaceae	0	0	0	0
Pseudomonas brassicacearum	Pseudomonas	Pseudomonadaceae	0	0	0	0
Pseudomonas chlororaphis	Pseudomonas	Pseudomonadaceae	2	0	0	0
Pseudomonas cichorii	Pseudomonas	Pseudomonadaceae	0	0	0	0
Pseudomonas costantinii	Pseudomonas	Pseudomonadaceae	0	0	0	0
Pseudomonas cremoricolorata	Pseudomonas	Pseudomonadaceae	0	0	0	0
Pseudomonas denitrificans	Pseudomonas	Pseudomonadaceae	1	0	0	2
Pseudomonas entomophila	Pseudomonas	Pseudomonadaceae	0	0	2	1
Pseudomonas extremaustralis	Pseudomonas	Pseudomonadaceae	0	0	0	2
Pseudomonas fluorescens	Pseudomonas	Pseudomonadaceae	1	5	1	2
Pseudomonas fulva	Pseudomonas	Pseudomonadaceae	4	0	0	0
Pseudomonas knackmussii	Pseudomonas	Pseudomonadaceae	0	0	0	0
Pseudomonas mandelii	Pseudomonas	Pseudomonadaceae	0	0	0	0
Pseudomonas marginalis	Pseudomonas	Pseudomonadaceae	0	0	0	0
Pseudomonas mendocina	Pseudomonas	Pseudomonadaceae	2	1	0	1
Pseudomonas mosselii	Pseudomonas	Pseudomonadaceae	15	4	2	1
Pseudomonas parafulva	Pseudomonas	Pseudomonadaceae	4	0	0	0
Pseudomonas poae	Pseudomonas	Pseudomonadaceae	0	0	0	0
Pseudomonas protegens	Pseudomonas	Pseudomonadaceae	0	0	0	0
Pseudomonas pseudoalcaligenes	Pseudomonas	Pseudomonadaceae	2	0	0	0
Pseudomonas putida	Pseudomonas	Pseudomonadaceae	7	4	0	1

Pseudomonas resinovorans	Pseudomonas	Pseudomonadaceae	0	0	0	0
Pseudomonas rhizosphaerae	Pseudomonas	Pseudomonadaceae	1	0	0	0
Pseudomonas savastanoi	Pseudomonas	Pseudomonadaceae	0	0	0	0
Pseudomonas simiae	Pseudomonas	Pseudomonadaceae	1	5	1	0
Pseudomonas sp. 12M76_air	Pseudomonas	Pseudomonadaceae	0	0	0	1
Pseudomonas sp. FGI182	Pseudomonas	Pseudomonadaceae	0	0	1	1
Pseudomonas sp. GP32	Pseudomonas	Pseudomonadaceae	0	0	0	0
Pseudomonas sp. Hx2	Pseudomonas	Pseudomonadaceae	0	0	0	0
Pseudomonas sp. MRSN12121	Pseudomonas	Pseudomonadaceae	0	0	1	0
Pseudomonas sp. MT-1	Pseudomonas	Pseudomonadaceae	0	0	0	0
Pseudomonas sp. OM2164	Pseudomonas	Pseudomonadaceae	0	0	0	1
Pseudomonas sp. StFLB209	Pseudomonas	Pseudomonadaceae	0	0	0	0
Pseudomonas sp. TKP	Pseudomonas	Pseudomonadaceae	3	3	3	2
Pseudomonas sp. USM7-7	Pseudomonas	Pseudomonadaceae	0	0	0	0
Pseudomonas sp. UW4	Pseudomonas	Pseudomonadaceae	0	1	0	0
Pseudomonas sp. VLB120	Pseudomonas	Pseudomonadaceae	0	0	0	0
Pseudomonas sp. WCS374	Pseudomonas	Pseudomonadaceae	2	0	0	2
Pseudomonas sp. Y2	Pseudomonas	Pseudomonadaceae	0	0	0	0
Pseudomonas sp. ZM1	Pseudomonas	Pseudomonadaceae	0	0	0	0
Pseudomonas stutzeri	Pseudomonas	Pseudomonadaceae	11	6	4	5
Pseudomonas synxantha	Pseudomonas	Pseudomonadaceae	0	0	0	0
Pseudomonas syringae	Pseudomonas	Pseudomonadaceae	3	0	0	0
Pseudomonas syringae group genom	Pseudomonas	Pseudomonadaceae	0	0	0	0
Pseudomonas taetrolens	Pseudomonas	Pseudomonadaceae	0	0	0	0
*	*	Pseudonocardiaceae	3	0	2	0
Actinosynnema mirum	Actinosynnema	Pseudonocardiaceae	6	0	1	0
*	Amycolatopsis	Pseudonocardiaceae	3	0	1	0
Amycolatopsis eurytherma	Amycolatopsis	Pseudonocardiaceae	0	0	0	1
Amycolatopsis japonica	Amycolatopsis	Pseudonocardiaceae	3	1	0	0
Amycolatopsis lurida	Amycolatopsis	Pseudonocardiaceae	0	0	1	0
Amycolatopsis mediterranei	Amycolatopsis	Pseudonocardiaceae	3	0	2	0
Amycolatopsis methanolica	Amycolatopsis	Pseudonocardiaceae	10	1	0	1
Amycolatopsis orientalis	Amycolatopsis	Pseudonocardiaceae	1	0	0	0
Kutzneria albida	Kutzneria	Pseudonocardiaceae	2	0	0	0
Pseudonocardia dioxanivorans	Pseudonocardia	Pseudonocardiaceae	5	4	1	1
Saccharomonospora viridis	Saccharomonospora	Pseudonocardiaceae	0	0	0	0
Saccharopolyspora erythraea	Saccharopolyspora	Pseudonocardiaceae	2	2	3	0
Saccharothrix espanaensis	Saccharothrix	Pseudonocardiaceae	0	0	1	0
Thermobispora bispora	Thermobispora	Pseudonocardiaceae	3	0	3	0
*	*	Rhizobiaceae	0	0	0	2
Agrobacterium fabrum	Agrobacterium	Rhizobiaceae	4	0	0	0
Agrobacterium tumefaciens	Agrobacterium	Rhizobiaceae	0	0	0	0
Agrobacterium vitis	Agrobacterium	Rhizobiaceae	5	0	0	0
Ensifer adhaerens	Ensifer	Rhizobiaceae	0	0	1	0
Neorhizobium galegae	Neorhizobium	Rhizobiaceae	1	0	0	0
*	Rhizobium	Rhizobiaceae	1	0	0	0
Rhizobium etli	Rhizobium	Rhizobiaceae	5	1	0	0
Rhizobium gallicum	Rhizobium	Rhizobiaceae	0	0	1	1
Rhizobium leguminosarum	Rhizobium	Rhizobiaceae	1	3	5	1
Rhizobium sp.	Rhizobium	Rhizobiaceae	0	0	2	0
Rhizobium sp. IRBG74	Rhizobium	Rhizobiaceae	0	1	0	0
Rhizobium sp. LPU83	Rhizobium	Rhizobiaceae	1	0	0	0
Rhizobium tropici	Rhizobium	Rhizobiaceae	0	0	1	0
Sinorhizobium fredii	Sinorhizobium	Rhizobiaceae	1	0	0	1
Sinorhizobium medicae	Sinorhizobium	Rhizobiaceae	0	0	0	0
*	*	Rhodobacteraceae	5	4	0	1
Celeribacter indicus	Celeribacter	Rhodobacteraceae	11	0	0	1
Dinoroseobacter shibae	Dinoroseobacter	Rhodobacteraceae	2	0	0	0
Jannaschia sp. CCS1	Jannaschia	Rhodobacteraceae	0	0	0	1
Ketogulonicigenium vulgare	Ketogulonicigenium	Rhodobacteraceae	2	0	0	0
Leisingera methylohalidivorans	Leisingera	Rhodobacteraceae	0	0	0	0
Octadecabacter antarcticus	Octadecabacter	Rhodobacteraceae	0	0	0	0
Octadecabacter arcticus	Octadecabacter	Rhodobacteraceae	0	0	2	0
*	Paracoccus	Rhodobacteraceae	1	0	0	0
Paracoccus aestuarii	Paracoccus	Rhodobacteraceae	0	1	0	0
Paracoccus aminophilus	Paracoccus	Rhodobacteraceae	7	3	3	0
Paracoccus denitrificans	Paracoccus	Rhodobacteraceae	24	3	5	10
Paracoccus marcusii	Paracoccus	Rhodobacteraceae	0	0	0	1
Paracoccus sp. 32d	Paracoccus	Rhodobacteraceae	0	0	0	0
Phaeobacter gallaeciensis	Phaeobacter	Rhodobacteraceae	2	0	0	0
*	Rhodobacter	Rhodobacteraceae	1	0	0	0
Rhodobacter capsulatus	Rhodobacter	Rhodobacteraceae	4	0	0	1
Rhodobacter sphaeroides	Rhodobacter	Rhodobacteraceae	11	0	3	1
Roseibacterium elongatum	Roseibacterium	Rhodobacteraceae	5	0	0	0
Roseobacter denitrificans	Roseobacter	Rhodobacteraceae	0	0	0	0
Roseobacter litoralis	Roseobacter	Rhodobacteraceae	2	0	0	0
Ruegeria pomeroyi	Ruegeria	Rhodobacteraceae	0	1	3	0
Ruegeria sp. TM1040	Ruegeria	Rhodobacteraceae	1	0	2	0
Parvibaculum lavamentivorans	Parvibaculum	Rhodobiaceae	0	0	0	0
Aromatoleum aromaticum	Aromatoleum	Rhodocyclaceae	0	1	0	0
Azoarcus sp. BH72	Azoarcus	Rhodocyclaceae	3	0	0	1
Azoarcus sp. KH32C	Azoarcus	Rhodocyclaceae	1	0	0	0

Azospira oryzae	Azospira	Rhodocyclaceae	0	1	0	0
Dechloromonas aromatica	Dechloromonas	Rhodocyclaceae	0	0	0	0
Thauera sp. MZ1T	Thauera	Rhodocyclaceae	2	0	0	0
*	Azospirillum	Rhodospirillaceae	0	0	0	0
Azospirillum brasilense	Azospirillum	Rhodospirillaceae	0	0	0	0
Azospirillum lipoferum	Azospirillum	Rhodospirillaceae	2	2	3	0
Magnetospirillum magneticum	Magnetospirillum	Rhodospirillaceae	0	0	0	0
Rhodospirillum centenum	Rhodospirillum	Rhodospirillaceae	0	0	0	0
*	Alistipes	Rikenellaceae	0	0	0	0
Alistipes finegoldii	Alistipes	Rikenellaceae	1	0	0	0
Alistipes shahii	Alistipes	Rikenellaceae	6	0	0	0
Mucinivorans hirudinis	Mucinivorans	Rikenellaceae	0	0	0	0
Calothrix sp. PCC 7507	Calothrix	Rivulariaceae	0	0	0	0
Roseiflexus sp. RS-1	Roseiflexus	Roseiflexaceae	0	0	0	0
Rubrobacter radiotolerans	Rubrobacter	Rubrobacteraceae	4	0	0	0
Rubrobacter xylanophilus	Rubrobacter	Rubrobacteraceae	2	0	0	0
Ethanoligenens harbinense	Ethanoligenens	Ruminococcaceae	0	0	0	0
Faecalibacterium prausnitzii	Faecalibacterium	Ruminococcaceae	49	5	3	1
[Eubacterium] siraeum	Ruminiclostridium	Ruminococcaceae	5	0	1	1
*	Ruminococcus	Ruminococcaceae	3	0	0	0
Ruminococcus bicirculans	Ruminococcus	Ruminococcaceae	5	0	0	0
Ruminococcus bromii	Ruminococcus	Ruminococcaceae	2	2	0	1
Ruminococcus champanellensis	Ruminococcus	Ruminococcaceae	1	0	0	0
Ruminococcus sp. SR1/5	Ruminococcus	Ruminococcaceae	11	0	3	0
Sanguibacter keddieii	Sanguibacter	Sanguibacteraceae	10	4	3	0
*	Shewanella	Shewanellaceae	0	0	0	0
Shewanella colwelliana	Shewanella	Shewanellaceae	0	0	0	0
Shewanella oneidensis	Shewanella	Shewanellaceae	3	0	0	0
Shewanella pealeana	Shewanella	Shewanellaceae	0	0	0	0
Shewanella sp. ANA-3	Shewanella	Shewanellaceae	2	0	0	0
Shewanella sp. W3-18-1	Shewanella	Shewanellaceae	2	0	0	0
Sphaerobacter thermophilus	Sphaerobacter	Sphaerobacteraceae	1	0	1	0
*	*	Sphingobacteriaceae	0	0	1	0
Pedobacter heparinus	Pedobacter	Sphingobacteriaceae	1	0	0	0
Pedobacter saltans	Pedobacter	Sphingobacteriaceae	1	0	0	3
Sphingobacterium sp. 21	Sphingobacterium	Sphingobacteriaceae	0	0	0	0
Sphingobacterium sp. ML3W	Sphingobacterium	Sphingobacteriaceae	8	0	0	0
Sphingobacterium sp. PM2-P1-29	Sphingobacterium	Sphingobacteriaceae	0	1	0	4
*	*	Sphingomonadaceae	5	1	0	0
*	Novosphingobium	Sphingomonadaceae	0	2	0	0
Novosphingobium aromaticivorans	Novosphingobium	Sphingomonadaceae	4	2	0	0
Novosphingobium pentaromativorans	Novosphingobium	Sphingomonadaceae	3	0	0	1
Novosphingobium sp. PP1Y	Novosphingobium	Sphingomonadaceae	0	0	0	0
*	Sphingobium	Sphingomonadaceae	0	0	0	0
Sphingobium chlorophenolicum	Sphingobium	Sphingomonadaceae	3	0	1	2
Sphingobium japonicum	Sphingobium	Sphingomonadaceae	10	1	2	0
Sphingobium sp. SYK-6	Sphingobium	Sphingomonadaceae	0	1	1	0
Sphingobium yanoikuyae	Sphingobium	Sphingomonadaceae	0	0	0	0
*	Sphingomonas	Sphingomonadaceae	0	0	0	0
Sphingomonas sanxanigenens	Sphingomonas	Sphingomonadaceae	2	0	0	1
Sphingomonas sp. 452	Sphingomonas	Sphingomonadaceae	1	0	0	0
Sphingomonas sp. KA1	Sphingomonas	Sphingomonadaceae	0	0	0	0
Sphingomonas sp. MM-1	Sphingomonas	Sphingomonadaceae	5	0	0	1
Sphingomonas sp. PB304	Sphingomonas	Sphingomonadaceae	1	0	1	0
Sphingomonas taxi	Sphingomonas	Sphingomonadaceae	4	2	3	2
Sphingomonas wittichii	Sphingomonas	Sphingomonadaceae	3	0	2	0
*	Sphingopyxis	Sphingomonadaceae	0	1	1	0
Sphingopyxis alaskensis	Sphingopyxis	Sphingomonadaceae	5	0	2	0
Sphingopyxis sp. Kp5.2	Sphingopyxis	Sphingomonadaceae	9	0	2	0
Zymomonas mobilis	Zymomonas	Sphingomonadaceae	1	0	0	0
Spirochaeta thermophila	Spirochaeta	Spirochaetaceae	0	0	0	0
Treponema primitia	Treponema	Spirochaetaceae	1	0	0	0
Treponema sp. OMZ 838	Treponema	Spirochaetaceae	2	0	0	0
Treponema succinifaciens	Treponema	Spirochaetaceae	2	0	0	1
Macrococcus caseolyticus	Macrococcus	Staphylococcaceae	8	1	2	0
*	Staphylococcus	Staphylococcaceae	5	0	4	2
Staphylococcus aureus	Staphylococcus	Staphylococcaceae	4	2	3	0
Staphylococcus epidermidis	Staphylococcus	Staphylococcaceae	10	7	1	2
Staphylococcus haemolyticus	Staphylococcus	Staphylococcaceae	0	1	1	0
Staphylococcus hominis	Staphylococcus	Staphylococcaceae	0	0	0	0
Staphylococcus lugdunensis	Staphylococcus	Staphylococcaceae	2	0	0	1
Staphylococcus pasteuri	Staphylococcus	Staphylococcaceae	1	0	0	7
Staphylococcus saprophyticus	Staphylococcus	Staphylococcaceae	7	0	0	0
Staphylococcus warneri	Staphylococcus	Staphylococcaceae	0	0	0	0
Staphylococcus xylosus	Staphylococcus	Staphylococcaceae	7	2	1	4
*	*	Streptococcaceae	0	1	0	1
Lactococcus garvieae	Lactococcus	Streptococcaceae	1	0	0	0
Lactococcus lactis	Lactococcus	Streptococcaceae	0	1	0	3
*	Streptococcus	Streptococcaceae	66	9	14	11
Streptococcus agalactiae	Streptococcus	Streptococcaceae	2	0	0	2
Streptococcus alactolyticus	Streptococcus	Streptococcaceae	3	0	1	0
Streptococcus anginosus	Streptococcus	Streptococcaceae	0	0	0	1

Streptococcus constellatus	Streptococcus	Streptococcaceae	1	0	0	0
Streptococcus dysgalactiae	Streptococcus	Streptococcaceae	1	1	1	0
Streptococcus equi	Streptococcus	Streptococcaceae	2	0	0	0
Streptococcus equinus	Streptococcus	Streptococcaceae	0	3	0	0
Streptococcus gallolyticus	Streptococcus	Streptococcaceae	11	3	3	2
Streptococcus gordonii	Streptococcus	Streptococcaceae	1	0	0	2
Streptococcus infantarius	Streptococcus	Streptococcaceae	11	3	0	13
Streptococcus iniae	Streptococcus	Streptococcaceae	1	0	0	0
Streptococcus intermedius	Streptococcus	Streptococcaceae	0	0	0	0
Streptococcus lutetiensis	Streptococcus	Streptococcaceae	10	0	1	1
Streptococcus macedonicus	Streptococcus	Streptococcaceae	11	1	1	3
Streptococcus mitis	Streptococcus	Streptococcaceae	0	0	0	0
Streptococcus mutans	Streptococcus	Streptococcaceae	5	0	3	0
Streptococcus oligofermentans	Streptococcus	Streptococcaceae	2	0	0	0
Streptococcus oralis	Streptococcus	Streptococcaceae	2	6	0	2
Streptococcus parasanguinis	Streptococcus	Streptococcaceae	0	0	0	0
Streptococcus parauberis	Streptococcus	Streptococcaceae	2	0	4	0
Streptococcus pasteurianus	Streptococcus	Streptococcaceae	5	3	0	1
Streptococcus pneumoniae	Streptococcus	Streptococcaceae	7	1	0	3
Streptococcus pseudopneumoniae	Streptococcus	Streptococcaceae	4	3	0	0
Streptococcus pyogenes	Streptococcus	Streptococcaceae	5	0	2	0
Streptococcus salivarius	Streptococcus	Streptococcaceae	3	1	0	1
Streptococcus sanguinis	Streptococcus	Streptococcaceae	1	1	0	0
Streptococcus sp. HZ3	Streptococcus	Streptococcaceae	0	0	0	0
Streptococcus sp. VT 162	Streptococcus	Streptococcaceae	5	2	5	0
Streptococcus suis	Streptococcus	Streptococcaceae	13	6	3	5
Streptococcus thermophilus	Streptococcus	Streptococcaceae	9	0	0	0
Streptococcus uberis	Streptococcus	Streptococcaceae	0	1	0	0
*	*	Streptomycetaceae	0	0	1	0
Kitasatospora setae	Kitasatospora	Streptomycetaceae	2	0	1	1
*	Streptomyces	Streptomycetaceae	21	6	3	7
Streptomyces albulus	Streptomyces	Streptomycetaceae	1	0	0	0
Streptomyces albus	Streptomyces	Streptomycetaceae	9	0	1	0
Streptomyces ambofaciens	Streptomyces	Streptomycetaceae	1	0	0	0
Streptomyces antibioticus	Streptomyces	Streptomycetaceae	0	0	0	0
Streptomyces autolyticus	Streptomyces	Streptomycetaceae	0	0	1	0
Streptomyces avermitilis	Streptomyces	Streptomycetaceae	0	9	1	0
Streptomyces bingchenggensis	Streptomyces	Streptomycetaceae	0	1	0	0
Streptomyces cattleya	Streptomyces	Streptomycetaceae	2	3	0	0
Streptomyces coelicolor	Streptomyces	Streptomycetaceae	2	0	0	0
Streptomyces collinus	Streptomyces	Streptomycetaceae	2	0	0	0
Streptomyces cyaneogriseus	Streptomyces	Streptomycetaceae	10	1	0	0
Streptomyces davawensis	Streptomyces	Streptomycetaceae	0	3	1	0
Streptomyces fulvissimus	Streptomyces	Streptomycetaceae	0	0	2	0
Streptomyces glaucescens	Streptomyces	Streptomycetaceae	1	0	0	1
Streptomyces griseus	Streptomyces	Streptomycetaceae	0	0	0	0
Streptomyces humidus	Streptomyces	Streptomycetaceae	0	0	0	0
Streptomyces hygroscopicus	Streptomyces	Streptomycetaceae	1	0	0	0
Streptomyces iranensis	Streptomyces	Streptomycetaceae	3	1	0	0
Streptomyces nodosus	Streptomyces	Streptomycetaceae	0	0	0	2
Streptomyces peucetius	Streptomyces	Streptomycetaceae	0	0	0	0
Streptomyces scabiei	Streptomyces	Streptomycetaceae	3	0	0	0
Streptomyces sp. 769	Streptomyces	Streptomycetaceae	7	0	0	0
Streptomyces sp. PAMC26508	Streptomyces	Streptomycetaceae	0	0	0	0
Streptomyces sp. PGA64	Streptomyces	Streptomycetaceae	1	0	0	0
Streptomyces sp. SirexAA-E	Streptomyces	Streptomycetaceae	8	0	1	0
Streptomyces venezuelae	Streptomyces	Streptomycetaceae	1	0	0	0
Streptomyces vietnamensis	Streptomyces	Streptomycetaceae	2	0	0	0
Streptomyces violaceusniger	Streptomyces	Streptomycetaceae	0	0	0	0
Nonomuraea sp. ATCC 39727	Nonomuraea	Streptosporangiaceae	0	0	2	0
Streptosporangium roseum	Streptosporangium	Streptosporangiaceae	0	0	0	2
Symbiobacterium thermophilum	Symbiobacterium	Symbiobacteriaceae	0	0	0	0
Syntrophobacter fumaroxidans	Syntrophobacter	Syntrophobacteraceae	2	0	0	0
Thermus oshimai	Thermus	Thermaceae	0	0	0	0
Moorella thermoacetica	Moorella	Thermoanaerobactera	0	0	2	0
Thermomonospora curvata	Thermomonospora	Thermomonosporacea	1	0	0	3
Truepera radiovictrix	Truepera	Trueperaceae	4	2	0	0
Tsukamurella paurometabola	Tsukamurella	Tsukamurellaceae	2	0	0	0
Megamonas hypermegale	Megamonas	Veillonellaceae	0	0	0	0
Megasphaera elsdenii	Megasphaera	Veillonellaceae	0	0	0	1
Mitsuokella multacida	Mitsuokella	Veillonellaceae	0	2	0	0
Selenomonas ruminantium	Selenomonas	Veillonellaceae	0	1	0	0
Selenomonas sputigena	Selenomonas	Veillonellaceae	0	0	0	0
Veillonella parvula	Veillonella	Veillonellaceae	4	2	0	5
Akkermansia muciniphila	Akkermansia	Verrucomicrobiaceae	0	0	0	0
*	Vibrio	Vibrionaceae	3	0	0	0
Vibrio nigripulchritudo	Vibrio	Vibrionaceae	2	0	0	0
Vibrio tasmaniensis	Vibrio	Vibrionaceae	0	0	2	0
Vibrio tubiashii	Vibrio	Vibrionaceae	0	0	0	0
Azorhizobium caulinodans	Azorhizobium	Xanthobacteraceae	1	0	0	0
Starkeya novella	Starkeya	Xanthobacteraceae	3	0	2	1
Xanthobacter autotrophicus	Xanthobacter	Xanthobacteraceae	0	1	0	0

*	*	Xanthomonadaceae	4	0	3	1
Dyella japonica	Dyella	Xanthomonadaceae	3	0	0	1
Dyella jiangningensis	Dyella	Xanthomonadaceae	0	0	0	0
Frateuria aurantia	Frateuria	Xanthomonadaceae	0	0	0	0
Lysobacter antibioticus	Lysobacter	Xanthomonadaceae	0	0	0	0
Lysobacter sp. ATCC 53042	Lysobacter	Xanthomonadaceae	0	0	0	0
Pseudoxanthomonas spadix	Pseudoxanthomonas	Xanthomonadaceae	1	0	0	0
Pseudoxanthomonas suwonensis	Pseudoxanthomonas	Xanthomonadaceae	0	2	0	2
Rhodanobacter denitrificans	Rhodanobacter	Xanthomonadaceae	7	0	6	3
*	Stenotrophomonas	Xanthomonadaceae	4	0	1	0
Stenotrophomonas maltophilia	Stenotrophomonas	Xanthomonadaceae	3	0	0	6
Stenotrophomonas rhizophila	Stenotrophomonas	Xanthomonadaceae	6	1	0	0
*	Xanthomonas	Xanthomonadaceae	1	0	1	0
Xanthomonas albilineans	Xanthomonas	Xanthomonadaceae	0	0	0	0
Xanthomonas campestris	Xanthomonas	Xanthomonadaceae	1	0	2	0
Xanthomonas oryzae	Xanthomonas	Xanthomonadaceae	0	0	0	0
Xanthomonas sacchari	Xanthomonas	Xanthomonadaceae	6	2	2	1
*	*		0	6	0	0
Candidatus Accumulibacter phosphat	Candidatus Accumulibacter		1	0	0	0
Chamaesiphon minutus	Chamaesiphon		1	0	0	0
Chroococcidiopsis thermalis	Chroococcidiopsis		6	3	0	1
*	Cyanothece		0	1	0	0
Cyanothece sp. ATCC 51142	Cyanothece		0	0	0	0
Cyanothece sp. PCC 7425	Cyanothece		0	0	0	0
*	Exiguobacterium		1	0	0	0
Exiguobacterium antarcticum	Exiguobacterium		0	0	0	2
Exiguobacterium sibiricum	Exiguobacterium		29	2	2	4
Exiguobacterium sp. AT1b	Exiguobacterium		2	0	0	0
Exiguobacterium sp. MH3	Exiguobacterium		7	2	0	3
Exiguobacterium sp. N139	Exiguobacterium		0	0	0	0
Fimbriimonas ginsengisoli	Fimbriimonas		1	0	0	0
Fischerella sp. MV11	Fischerella		0	0	0	0
Gloeobacter kilaueensis	Gloeobacter		1	0	0	1
Gloeocapsa sp. PCC 7428	Gloeocapsa		4	1	0	0
Gottschalkia acidurici	Gottschalkia		0	0	0	0
Leptolyngbya boryana	Leptolyngbya		4	3	3	1
Leptothrix cholodnii	Leptothrix		3	1	0	0
Methylibium petroleiphilum	Methylibium		3	1	0	1
Microcoleus sp. PCC 7113	Microcoleus		0	1	0	0
Oscillatoria acuminata	Oscillatoria		0	0	0	0
Oscillatoria nigro-viridis	Oscillatoria		9	0	1	1
Pleurocapsa minor	Pleurocapsa		1	0	0	0
Polymorphum gilvum	Polymorphum		6	0	1	0
Rubrivivax gelatinosus	Rubrivivax		7	2	0	0
Stanieria cyanosphaera	Stanieria		0	0	0	0
*	Synechococcus		0	1	0	0
Synechococcus sp. CC9605	Synechococcus		5	0	0	0
Synechococcus sp. PCC 7002	Synechococcus		0	0	0	0
Synechococcus sp. PCC 7502	Synechococcus		1	0	0	0
Thermosynechococcus sp. NK55a	Thermosynechococcus		0	0	0	0
*	Thiomonas		0	0	0	0
Thiomonas arsenitoxydans	Thiomonas		0	2	0	0
Thiomonas intermedia	Thiomonas		0	2	0	0
Burkholderiales bacterium GJ-E10			1	0	0	0
Candidatus Baumannia cicadellinicola			0	0	0	1
Candidatus Saccharibacteria bacterium RAAC3_TM7_1			0	0	0	1
Gemmatimonadetes bacterium KBS708			1	0	1	0
Pseudomonas sp.			1	0	0	1
Theonella swinhoei bacterial symbiont clone pSW1H8			1	0	0	0
beta proteobacterium CB			0	0	0	0
butyrate-producing bacterium SM4/1			0	0	0	0
butyrate-producing bacterium SS3/4			8	3	0	0
butyrate-producing bacterium SSC/2			4	0	0	1
cyanobacterium Atacama7			2	0	0	0
cyanobacterium endosymbiont of Epithemia turgida			0	0	0	0

Table D. Non-Chordate Eukaryotic Reads Detected by SURPI for the Koch's Postulate SC lysates.

Species	Genus	Family	Koch'sPostulate Step 2: SC lysate (dpi 4)	Koch's Postulate Step 4: SC lysate (dpi 3)	Koch's Postulate Step 4: SC lysate (dpi 4)	Koch's Postulate Step 4: SC lysate (dpi 5)
*	*	*	336	235	410	365
*	Chelidonura	Aglajidae	0	0	0	3
Albugo laibachii	Albugo	Albuginaceae	0	0	0	0
Angiostrongylus cantonensis	Angiostrongylus	Angiostrongylidae	0	0	0	0
Anisakis simplex	Anisakis	Anisakidae	0	0	0	0
Aplysia californica	Aplysia	Aplysiidae	0	0	0	0
*	*	Arthrodermataceae	0	0	0	0
*	Aspergillus	Aspergillaceae	1	0	0	0
Aspergillus niger	Aspergillus	Aspergillaceae	1	0	0	0
Neosartorya fischeri	Neosartorya	Aspergillaceae	0	0	2	0
Auricularia delicata	Auricularia	Auriculariaceae	1	0	0	0
*	Babesia	Babesiidae	0	0	0	0
Babesia microti	Babesia	Babesiidae	11	4	10	11
Heterobasidion irregulare	Heterobasidion	Bondarzewiaceae	1	0	0	0
Monosiga brevicollis	Monosiga	Codonosigidae	0	0	0	0
Cyclophorus consociatus	Cyclophorus	Cyclophoridae	0	0	0	0
Cyphellophora europaea	Cyphellophora	Cyphellophoraceae	75	25	30	18
Candida parapsilosis	Candida	Debaryomycetaceae	0	0	1	0
Debaryomyces hansenii	Debaryomyces	Debaryomycetaceae	1	0	0	0
Lodderomyces elongisporus	Lodderomyces	Debaryomycetaceae	0	5	0	0
Diphyllobothrium latum	Diphyllobothrium	Diphyllobothriidae	0	0	0	0
Spirometra erinaceieuropaei	Spirometra	Diphyllobothriidae	1	0	0	0
Schmidtea mediterranea	Schmidtea	Dugesiidae	0	0	0	0
Echinostoma caproni	Echinostoma	Echinostomatidae	1	1	0	2
Nematostella vectensis	Nematostella	Edwardsiidae	0	0	0	0
Enterocytozoon bieneusi	Enterocytozoon	Enterocytozoonidae	0	0	0	0
Rhizophagus intraradices	Rhizophagus	Glomeraceae	0	0	0	0
Helobdella robusta	Helobdella	Glossiphoniidae	0	0	0	1
Gongylonema pulchrum	Gongylonema	Gongylonematidae	2	2	0	0
Haemonchus placei	Haemonchus	Haemonchidae	0	0	0	0
Nippostrongylus brasiliensis	Nippostrongylus	Heligmonellidae	0	0	0	0
Heligmosomoides polygyrus	Heligmosomoides	Heligmosomatidae	0	0	1	0
Capronia coronata	Capronia	Herpotrichiellaceae	0	0	0	0
Cladophialophora psammophila	Cladophialophora	Herpotrichiellaceae	2	0	0	0
Exophiala dermatitidis	Exophiala	Herpotrichiellaceae	0	0	0	0
Hydra vulgaris	Hydra	Hydridae	0	0	0	1
Hymenolepis diminuta	Hymenolepis	Hymenolepididae	0	0	1	0
Hymenolepis nana	Hymenolepis	Hymenolepididae	0	0	0	1
*	Leptosphaeria	Leptosphaeriaceae	0	0	0	0
Leptosphaeria biglobosa	Leptosphaeria	Leptosphaeriaceae	0	0	0	0
Leptosphaeria maculans	Leptosphaeria	Leptosphaeriaceae	0	0	0	0
Gaeumannomyces graminis	Gaeumannomyces	Magnaporthaceae	0	1	0	0
Malassezia globosa	Malassezia	Malasseziaceae	34	16	16	4
Malassezia sympodialis	Malassezia	Malasseziaceae	0	0	0	0
Clavispora lusitaniae	Clavispora	Metschnikowiaceae	0	0	0	0
*	Fusarium	Nectriaceae	0	3	0	0
Fusarium graminearum	Fusarium	Nectriaceae	0	0	0	1
Brugia pahangi	Brugia	Onchocercidae	0	0	0	1
Brugia timori	Brugia	Onchocercidae	1	1	0	0
Elaeophora elaphi	Elaeophora	Onchocercidae	1	1	3	1
Onchocerca flexuosa	Onchocerca	Onchocercidae	0	0	0	0
Onchocerca ochengi	Onchocerca	Onchocercidae	0	0	1	0
Wuchereria bancrofti	Wuchereria	Onchocercidae	0	1	1	0
Crassostrea gigas	Crassostrea	Ostreidae	3	1	1	0
Enterobius vermicularis	Enterobius	Oxyuridae	2	0	0	0
Syphacia muris	Syphacia	Oxyuridae	1	1	0	0
Parastagonospora nodorum	Parastagonospora	Phaeosphaeriaceae	0	0	0	0
Physella acuta	Physella	Physidae	0	0	0	0
Verticillium dahliae	Verticillium	Plectosphaerellaceae	0	1	0	0
*	*	Pleosporaceae	0	0	0	1
Bipolaris zeicola	Bipolaris	Pleosporaceae	2	0	0	0
Protopolystoma xenopodis	Protopolystoma	Polystomatidae	3	3	6	0
Caenorhabditis remanei	Caenorhabditis	Rhabditidae	0	2	0	0
Naumovozyma castellii	Naumovozyma	Saccharomycetaceae	0	0	0	0
Salpingoeca rosetta	Salpingoeca	Salpingoecidae	0	1	1	0
Aphanomyces euteiches	Aphanomyces	Saprolegniaceae	0	0	0	0
Neospora caninum	Neospora	Sarcocystidae	0	0	0	0
Schistosoma japonicum	Schistosoma	Schistosomatidae	1	0	4	10
Schistosoma mansoni	Schistosoma	Schistosomatidae	0	0	0	0
Schistosoma rodhaini	Schistosoma	Schistosomatidae	0	0	0	0
Trichobilharzia regenti	Trichobilharzia	Schistosomatidae	0	1	0	0
*	*	Sclerotiniaceae	0	0	0	0
Botrytis cinerea	Botrytis	Sclerotiniaceae	52	7	10	10
Patagoniobdella fraterna	Patagoniobdella	Semiscolecidae	0	0	0	1
Patagoniobdella variabilis	Patagoniobdella	Semiscolecidae	0	0	0	0
Soboliphyme baturini	Soboliphyme	Soboliphymatidae	0	1	0	1
Sordaria macrospora	Sordaria	Sordariaceae	0	0	0	0
Cylicostephanus goldi	Cylicostephanus	Strongylidae	0	0	3	0
Strongylus vulgaris	Strongylus	Strongylidae	0	1	0	0
Parastrongyloides trichosuri	Parastrongyloides	Strongyloididae	19	11	12	5
Taenia asiatica	Taenia	Taeniidae	0	0	0	0
Theileria parva	Theileria	Theileriidae	0	0	0	0
Toxocara canis	Toxocara	Toxocaridae	0	0	0	0
Trichinella spiralis	Trichinella	Trichinellidae	0	0	1	0
Leishmania major	Leishmania	Trypanosomatidae	0	0	0	0
Pseudozyma flocculosa	Pseudozyma	Ustilaginaceae	3	0	0	0
Sporisorium reilianum	Sporisorium	Ustilaginaceae	0	1	0	0
Acanthamoeba castellanii	Acanthamoeba		0	0	0	0
Aureococcus anophagefferens	Aureococcus		1	0	0	0
*	Entamoeba		0	0	0	0
*	Plasmodium		2	0	3	5
Plasmodium berghei	Plasmodium		10	19	27	9
Plasmodium chabaudi	Plasmodium		0	0	0	1
Rhodosporidium toruloides	Rhodosporidium		5	0	0	0
Rhodotorula glutinis	Rhodotorula		1	0	0	0
Rhodotorula taiwanensis	Rhodotorula		1	0	0	0
Tubulideres seminoli	Tubulideres		0	0	0	0
Wallemia sebi	Wallemia		4	1	2	1

Note: samples from an unrelated study of *Babesia microti* sequencing were presented on the same sequencing run; thus, reads aligning to *Babesia* were seen in all of the sequencing libraries
