# Supplementary material for: A mouse model of paralytic myelitis caused by enterovirus D68
Source: PLoS Pathog. 2017 Feb 23;13(2):e1006199. doi: 10.1371/journal.ppat.1006199 (PMC5322875; doi:10.1371/journal.ppat.1006199)
Supplement: S1 Table — On day post-injection (dpi) 6, mice were sacrificed, and muscle tissue of the injected leg was harvested for TCID50 analysis. At this time point, all MO/14-18947 mice were showing signs of paralysis in the injected limb, while none of the Fermon, Rhyne, or CA/14-4231 mice had signs of paralysis. Viral infection was detected within the muscle from mice injected with MO/14-18947, CA/14-4231, and Rhyne as calculated by TCID50/mg of muscle tissue. Fermon was not detected in the muscle tissue of any mouse. (PDF) [file ppat.1006199.s009.pdf]

Table S1

EV-D68 Strain	n	% with paralysis	Average log10(TCID50/mg) in muscle +/- SEM
**Fermon**	4	0%	not detectable
**Rhyne**	5	0%	3.2 +/- 0.1
**CA/14-4231**	4	0%	2.7 +/- 0.2
**MO/14-18947**	4	100%	2.6 +/- 0.3
